# Tension Remodeling Regulates Topological Transitions in Epithelial Tissues

**DOI:** 10.1103/prxlife.1.023006

**Published:** 2023-11-27

**Authors:** Fernanda Pérez-Verdugo, Shiladitya Banerjee

**Affiliations:** Department of Physics, Carnegie Mellon University, Pittsburgh, Pennsylvania 15213, USA

## Abstract

Cell neighbor exchanges play a critical role in regulating tissue fluidity during epithelial morphogenesis and repair. *In vivo*, these neighbor exchanges are often hindered by the formation of transiently stable fourfold vertices, which can develop into complex multicellular rosettes where five or more cell junctions meet. Despite their importance, the mechanical origins of multicellular rosettes have remained elusive, and current cellular models lack the ability to explain their formation and maintenance. Here we present a dynamic vertex model of epithelial tissues with strain-dependent tension remodeling and mechanical memory dissipation. We show that an increase in cell junction tension upon contraction and reduction in tension upon extension can stabilize higher-order vertices, temporarily stalling cell rearrangements. On the other hand, inducing mechanical memory dissipation via relaxation of junction strain and stress promotes the resolution of higher-order vertices, facilitating cell neighbor exchanges. We demonstrate that by tuning the rates of tension remodeling and mechanical memory dissipation, we can control topological transitions and tissue material properties, recapitulating complex cellular topologies seen in developing organisms.

## I. INTRODUCTION

Fluidization of epithelial tissues plays a vital role in coordinating large-scale structural changes in early development [1,2], wound healing [3], and collective cell migration [4–6]. While multiple cell-level mechanisms contribute to tissue fluidity, including cell migration, division, and death [7], cell neighbor exchanges are one of the most common drivers of tissue fluidity during morphogenesis [8,9]. During a neighbor exchange process occurring via a T1 transition, two cells in contact shrink their shared junction to a single point, forming a fourfold vertex. This fourfold vertex then extends into a new intercellular junction in a direction orthogonal to the contracting junction. While neighbor exchange processes rely on the instability of fourfold vertices, *in vivo* experiments showed that fourfold vertices can be stable for long times in developing tissues [10–13]. In particular, during axis elongation in *Drosophila*, vertices shared by four or more cells (termed rosettes) could persist for up to 15–40 min [11,13], stalling cell neighbor exchanges.

Experimental observations of controlled cell neighbor exchanges contrast with existing vertex models of epithelial tissues [14,15], where stationary fourfold vertices do not naturally arise and are energetically unstable [16]. Furthermore, experiments showed that fourfold and higher-order vertices often restore the original cell junction, resulting in a reversible T1 process [12,13,17]. By contrast, most theoretical studies treat the creation and resolution of fourfold vertices as an instantaneous and unidirectional event triggered by junctions contracting below a length threshold [18] or if neighbor exchange is energetically favorable [19,20]. Others have engineered the formation of higher-order vertices by *ad hoc* rules. For instance, Farhadifar *et al*. [21,22] enforced the creation of fourfold vertices by joining proximal threefold vertices and stalling their subsequent resolution. On the other hand, there have been recent theoretical efforts to understand the impact of noninstantaneous resolution of fourfold vertices and probabilistic T1 events [13,23–25]. However, these studies imposed the stalling of T1 events by *ad hoc* rules and they did not naturally arise from the underlying mechanics of the tissue.

To explain the physical origin of fourfold vertex stability and controlled T1 transitions in epithelial tissues, we extended the existing framework of vertex models [14,15,21] to incorporate dynamic tension remodeling and mechanical memory dissipation. In particular, the tension in intercellular junctions evolves in time due to changes in junctional strain above a threshold or in response to active fluctuations. We show that tension remodeling and mechanical memory dissipation lead to controlled cell neighbor exchanges such that T1 transitions are stalled when they are not energetically favorable. By tuning the rates of tension remodeling, we can control the probability of reversible and irreversible T1 transitions, as well as the timescale of stalling of fourfold vertices. While the mechanical stability of higher-order vertices relies on the ability of cellular junctions to remodel their tension in response to strain, their resolution requires timely dissipation of mechanical memory in the system. Therefore, transient stabilization of n-fold vertices (n>3) relies on mechanical memory dissipation, which could occur via relaxation of junctional tension, strain, or noise-induced tension fluctuations. In addition to regulating tissue topology and cell morphologies, tension remodeling rates also control the emergent material properties of the tissue. In particular, we show that by tuning the rates of tension remodeling, epithelial tissues can transition between solid and fluidlike phases with tunable rates of energy dissipation. Taken together, our theory and simulations uncover the mechanical requirements for controlled T1 transitions in epithelial tissues and elucidate the mechanics underlying solid-fluid transitions in epithelia.

## II. VERTEX MODEL WITH TENSION REMODELING

### A. Forces and equations of motion

To describe the dynamics of topological transitions in confluent tissues, we use the framework of the vertex model [14,21,26,27], where each cell is modeled as a two-dimensional polygon, with edges representing the cell-cell junctions and the vertices representing multicellular junctions. The overdamped dynamics of vertices are determined by a balance of forces between friction, cell elasticity, and active forces acting at intercellular junctions. The position $r_{i}$ of vertex i evolves in time as

(1) $$\mu \frac{\text{d}r_{i}}{\text{d}t}=-\frac{\partial E_{el}}{\partial r_{i}}+F_{i}^{act},$$

where μ is the vertex friction coefficient, $E_{el}=\sum_{\alpha }\;(K/2){\left(A_{\alpha }-A_{\alpha }^{0}\right)}^{2}$ penalizes changes in the area $A_{\alpha }$ of cell α, with respect to its target value $A_{\alpha }^{0}$, and K is the bulk elastic modulus. Active forces arise from actomyosin contractility $\Gamma_{a}$ in the cell cortex and tension T at intercellular junctions such that $F_{i}^{\text{act}}=-\sum_{\left\langle ij\right\rangle }\;\left(T_{ij}+\Gamma_{a}l_{ij}\right)\left(\partial l_{ij}/\partial r_{i}\right)$, where $T_{ij}$ is the tension on an edge connecting vertices i and j with length $l_{ij}$ [28,29]. Tension due to actomyosin contractility is proportional to junction length, qualitatively similar to perimeter-dependent contractility term in classical vertex models [14]. This captures the positive-feedback effect that myosin recruitment increases with increasing junction length [30]. Note that the force due to $\Gamma_{a}$ could also be interpreted as a conservative force arising from an energy term $\sum_{\left\langle ij\right\rangle }\;\Gamma_{a}l_{ij}^{2}/2$.

Several recent studies provided evidence that tension in epithelial cell junctions is not static but a dynamic quantity maintained by mechanochemical feedback processes [17,28,31–36]. We therefore model junctional tension as $T_{ij}(t)=\Lambda_{ij}(t)+\Delta \Lambda_{ij}(t)$, where $\Lambda_{ij}(t)$ is the deterministic part of the tension and $\Delta \Lambda_{ij}(t)$ represents stochastic fluctuations in tension. The dynamics of $\Lambda_{ij}$ is dependent on the junctional strain $\varepsilon_{ij}=\left(l_{ij}-l_{ij}^{0}\right)/l_{ij}^{0}$, where $l_{ij}^{0}$ is the junction rest length. Tension $\Lambda_{ij}$ evolves in time as

(2) $$\frac{\text{d}\Lambda_{ij}}{\text{d}t}=-\alpha \left(\varepsilon_{ij}\right)\left(l_{ij}-l_{ij}^{0}\right)-\frac{1}{\tau_{\Lambda }}\left(\Lambda_{ij}-\Lambda_{0}\right),$$

where the first term describes strain-dependent tension remodeling, as recently introduced by us [28] and tested experimentally [31,32,37], and the second term describes tension relaxation to a mean value $\Lambda_{0}$, which occurs over a longer characteristic timescale $\tau_{\Lambda }$. Motivated by recent experiments on single-junction mechanics [31,37], the rate of tension remodeling α (units of force per unit length per unit time) is defined as

(3) $$\alpha \left(\varepsilon_{ij}\right)=\left\{\begin{array}{ll} k_{C} & \text{ if }\varepsilon_{ij}<-\varepsilon_{c}\\ k_{E} & \text{ if }\varepsilon_{ij}>\varepsilon_{c}\\ 0 & \text{otherwise,} \end{array}\right.$$

where $\varepsilon_{c}$ is a threshold strain for junction remodeling. With positive $k_{E}$ and $k_{C}$, there is a negative-feedback effect such that tension increases upon contraction at a rate $k_{C}$ and reduces upon stretch at a rate $k_{E}$, consistent with experimental observations [31,32,35]. The threshold strain is motivated by optogenetics data on single-junction activation that show cellular junctions only remodel their length above a threshold contraction [28]. Tension remodeling above a critical strain threshold allows for irreversible junction deformation for sufficiently strong or sustained force [31].

Additionally, cellular junctions continuously relax strain at a rate $k_{L}$ such that the junction rest length approaches the current length as

(4) $$\frac{\text{d}l_{ij}^{0}}{\text{d}t}=-k_{L}\left(l_{ij}^{0}-l_{ij}\right).$$

Strain relaxation via rest length remodeling [28,38,39] is a natural consequence of turnover in strained actomyosin networks [40], where deformed filaments are replaced by unstrained ones. An important consequence of strain relaxation is that memory of prior deformations is erased over a timescale $k_{L}^{-1}$ such that long periods of contractions can remodel junctions only up to a limit, while pulsatile contractions with periods of rest enable irreversible deformations via ratcheting [28,31].

Finally, tension fluctuations $\Delta \Lambda_{ij}$ evolve according to an Ornstein-Uhlenbeck process as [3,17]

(5) $$\frac{\text{d}\Delta \Lambda_{ij}}{\text{d}t}=-\frac{1}{\tau }\Delta \Lambda_{ij}+\sqrt{2\sigma^{2}/\tau }\xi_{ij}(t),$$

where σ is the fluctuation amplitude, $\xi_{ij}(t)$ is a white Gaussian noise satisfying $\left\langle \xi_{ij}(t)\xi_{mn}\left(t^{\prime }\right)\right\rangle =\delta \left(t-t^{\prime }\right)\delta_{im}\delta_{jn}$, and τ is the persistence time of tension fluctuations. Our model thus considers three principal mechanisms for erasing the memory of the prior mechanical state, via tension relaxation at a rate $\tau_{\Lambda }^{-1}$, tension fluctuations of amplitude σ, and continuous strain relaxation at a rate $k_{L}$. As shown later, the transient stabilization of higher-order vertices is crucially dependent on the ability of tissues to dissipate mechanical memory.

### B. Mechanical stability and viscoelasticity of cell junctions

**FIG. 1.**
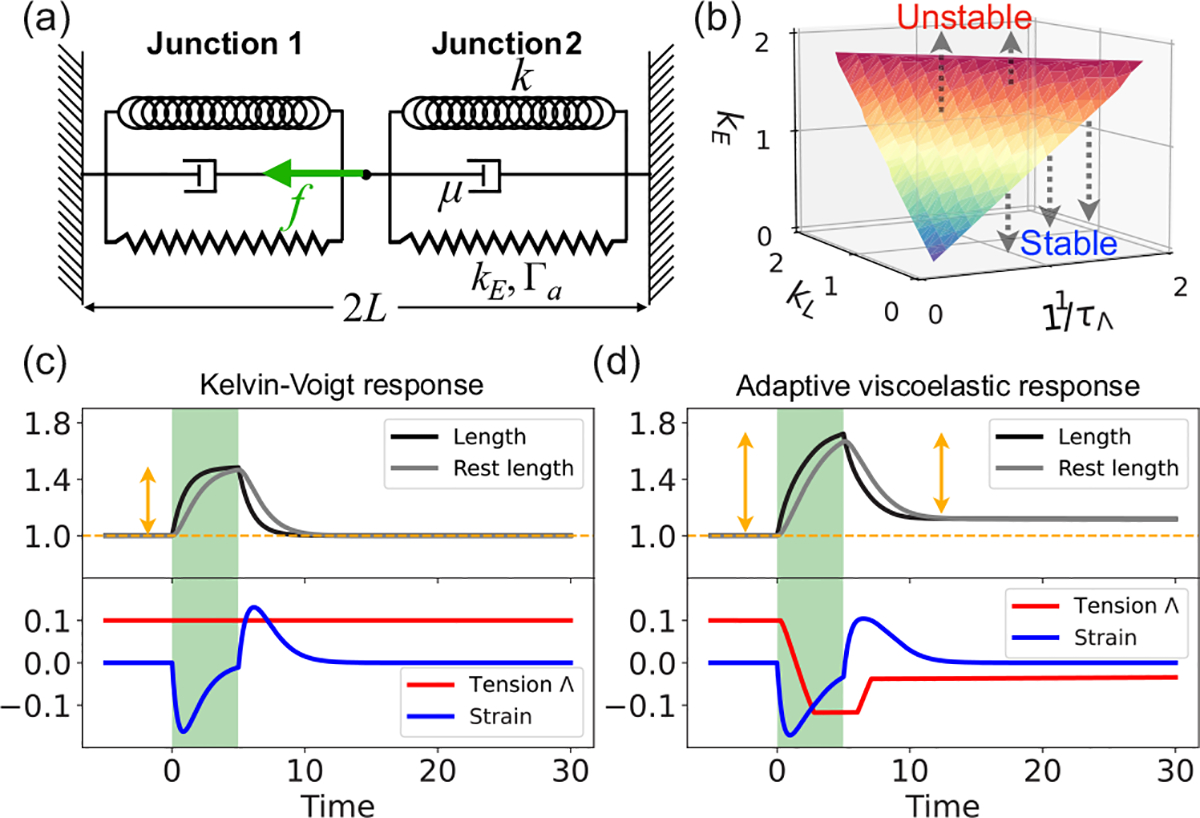
Junction stability and adaptive viscoelastic response. (a) Schematic of a simplified two-junction model under active contraction. (b) Phase diagram in the $\left(k_{E}=k_{c},\;k_{L},\;\tau_{\Lambda }^{-1}\right)$ plane, showing the critical surface separating stable and unstable regimes of the system. Color, from blue to red, represents the value of $k_{E}$. (c) and (d) Dynamical response of junction (2) length, rest length, strain, and tension Λ for (c) $k_{E}=k_{C}=0$ and (d) $k_{E}=k_{C}=0.5$. The parameters in (b)–(d) are $\Gamma_{a}=0.03,\;k_{L}=1$, and $\tau_{\Lambda }^{-1}=0.03$.

We begin by examining the mechanical response of individual junctions to contractile forces. To do this, we simplify the model by considering a one-dimensional variant of Eqs. (1)–(4), neglecting any stochastic fluctuations (as depicted in Fig. 1). This simplified model focuses on a two-junction system with varying lengths, denoted by $l_{1}(t)$ and $l_{2}(t)$, respectively. Each junction unit is comprised of an elastic element with a spring constant k and natural length L, connected in parallel to a dashpot with friction coefficient μ [Fig. 1(a)]. Additionally, an active elastic element with a rest length $l_{1,2}^{0}$ and contractility $\Gamma_{a}$ is connected in parallel to the dashpot and the spring. As previously described, the tension in the junction is remodeled at a rate $k_{E}$ under contraction and stretching, with an initial value of $\Lambda_{0}$ and a rest length $l^{0}$ that relaxes toward the current junction length with a rate $k_{L}$ In our analysis, we assume fixed boundary conditions, allowing only the middle vertex to move under applied forces.

To examine the mechanical stability of these junctions under applied forces, we derive a linearized system of equations for a perturbation $\delta X=(\delta \Gamma_{1},\delta \Gamma_{2},\delta l_{1}^{0},\delta l_{2}^{0},\delta l_{1})$ around the steady state as δẊ=AδX. The δX follows the dynamics described in Eqs. (1)–(4), with the elastic energy given by $E_{el}=\frac{k}{2}{\left(L-l_{1}\right)}^{2}+\frac{k}{2}{\left(L-l_{2}\right)}^{2}$. We nondimensionalize force scales by kL, length scales by L, and timescales by μ/k, setting k=L=μ=1. In this particular analysis, we assume that $\varepsilon_{c}=0$ such that even the slightest perturbation would induce junction tension remodeling at a rate $k_{E}$. We then numerically diagonalize the stability matrix 𝔸 for different values of the rates $k_{L}$ (rest length relaxation), $1/\tau_{\Lambda }$ (tension relaxation), and $k_{E}$ (tension remodeling). We find that the system is stable (maximum eigenvalue of A ⩽ 0) in the absence of tension remodeling. However, it becomes unstable at a critical value of $k_{E}=k_{E}^{*}$, where $k_{E}^{*}$ increases in conjunction with both $k_{L}$ and $1/\tau_{\Lambda }$ [Fig. 1(b)]. Physically, this instability would manifest as junction collapse.

The one-dimensional junction model reveals adaptive viscoelastic properties that are essential for understanding tissue-level mechanical response. In addition to elasticity and dissipation through friction, cell junctions have additional sources of dissipation through tension remodeling, tension relaxation, and strain relaxation. We therefore seek to analyze the viscoelastic response of individual junctions by performing a load-controlled tension test. Specifically, we apply a constant tension f in the middle vertex, for a time period of 5(μ/k) [Figs. 1(c) and 1(d)], and monitor the dynamics of tension and length in junction 2, both with [Fig. 1(c)] and without tension remodeling [Fig. 1(d)]. In the absence of tension remodeling $\left(k_{E}=k_{C}=0\right)$, we obtain $f=(1+\left.2\Gamma_{a}\right){\tilde{\varepsilon }}_{2}+\text{d}{\tilde{\varepsilon }}_{2}/\text{d}t$, with ${\tilde{\varepsilon }}_{2}=l_{2}(t)-1$. Hence, the system behaves like a Kelvin-Voigt viscoelastic solid. When $k_{E}>0$ the response during load is amplified, while the unloading behavior is dependent on tension relaxation rate $\tau_{\Lambda }^{-1}$. For $1/\tau_{\Lambda }\ll \mu /k$, the relaxation during unloading is slow, leading to a steady state with a longer equilibrium junction length $l_{2}>L$ [Fig. 1(d)]. For $1/\tau_{\Lambda }<\mu /k$, we observe an undershoot in the length dynamics $l_{2}(t)$ during recovery from load (figure not shown). For $1/\tau_{\Lambda }\gg \mu /k$, the system responds like a Kelvin-Voigt viscoelastic solid.

### C. Implementation of T1 transitions

With the model mechanics defined above, we now turn to describing the dynamics governing T1 topological transitions. To simulate a T1 transition, when a junction connected by two threefold vertices becomes shorter than a threshold length $l_{T_{1}}$, one of the vertices is removed while the other is transformed into a fourfold vertex, sustained by four shoulder junctions. During this process, each shoulder junction gains one-fourth of the deleted junction tension and conserves it until the fourfold vertex is resolved [41]. The latter is motivated by experimental observations of myosin II accumulation around junctions proximal to fourfold vertices [17]. We then create a new junction of length $l_{\text{birth}}=1.5l_{T_{1}}$ and tension $\Lambda_{\text{birth}}\sim \Lambda_{0}+\Gamma_{a}l_{birth}$ and attempt to resolve the fourfold vertex in two different directions, one along the original contracting junction (resulting in reversible T1) and the other approximately orthogonal to it (leading to neighbor exchange), as experimentally observed [10,17]. To decide the final resolution configuration we follow an approach previously introduced in Ref. [16]. If the force between the vertices of the newly created junction is attractive, then the fourfold vertex is considered stable and the T1 transition is stalled. Otherwise, the fourfold vertex is resolved in the direction with the largest separation force [16,17,42], resulting in reversible or irreversible T1 transitions (see the Supplemental Material [43] for details). As discussed later, the specific choice of T1 transition parameters, such as $\Lambda_{\text{birth}}$ and $l_{\text{birth}}$, as well as the choice of tension resetting rule does not influence our main conclusions on the formation and stability of fourfold vertices.

#### Higher-order vertex formation and resolution

The rules for fourfold vertex formation, as described above, can also be applied to the merging of a stable n-fold and a threefold vertex, allowing the possibility of an (n+1)-fold vertex in the tissue. During the creation of such an (n+1) fold vertex, each shoulder junction gains 1/(n+1) th of the tension of the deleted junction, as in the n=3 case previously described. However, a more general idea of the resolution directions is needed. Here we propose that an (n+1)-fold vertex can be resolved into an n-fold and a threefold vertex in n+1 possible directions given by $\left(R_{c}^{\alpha }-r_{n+1}\right)/\left|R_{c}^{\alpha }-r_{n+1}\right|$, where $R_{c}^{\alpha }$ is the center of one (α) of the n+1 cells surrounding the (n+1)-fold vertex with position $r_{n+1}$. See the Supplemental Material [43] for further details on n-fold vertices, Fig. S2 [43], and Movie 4 for a simulated tissue in which threefold, fourfold, and fivefold vertices are allowed. In the rest of this paper, we allow for only threefold and fourfold vertices.

## III. RESULTS

### A. Tension remodeling controls T1 transitions

**FIG. 2.**
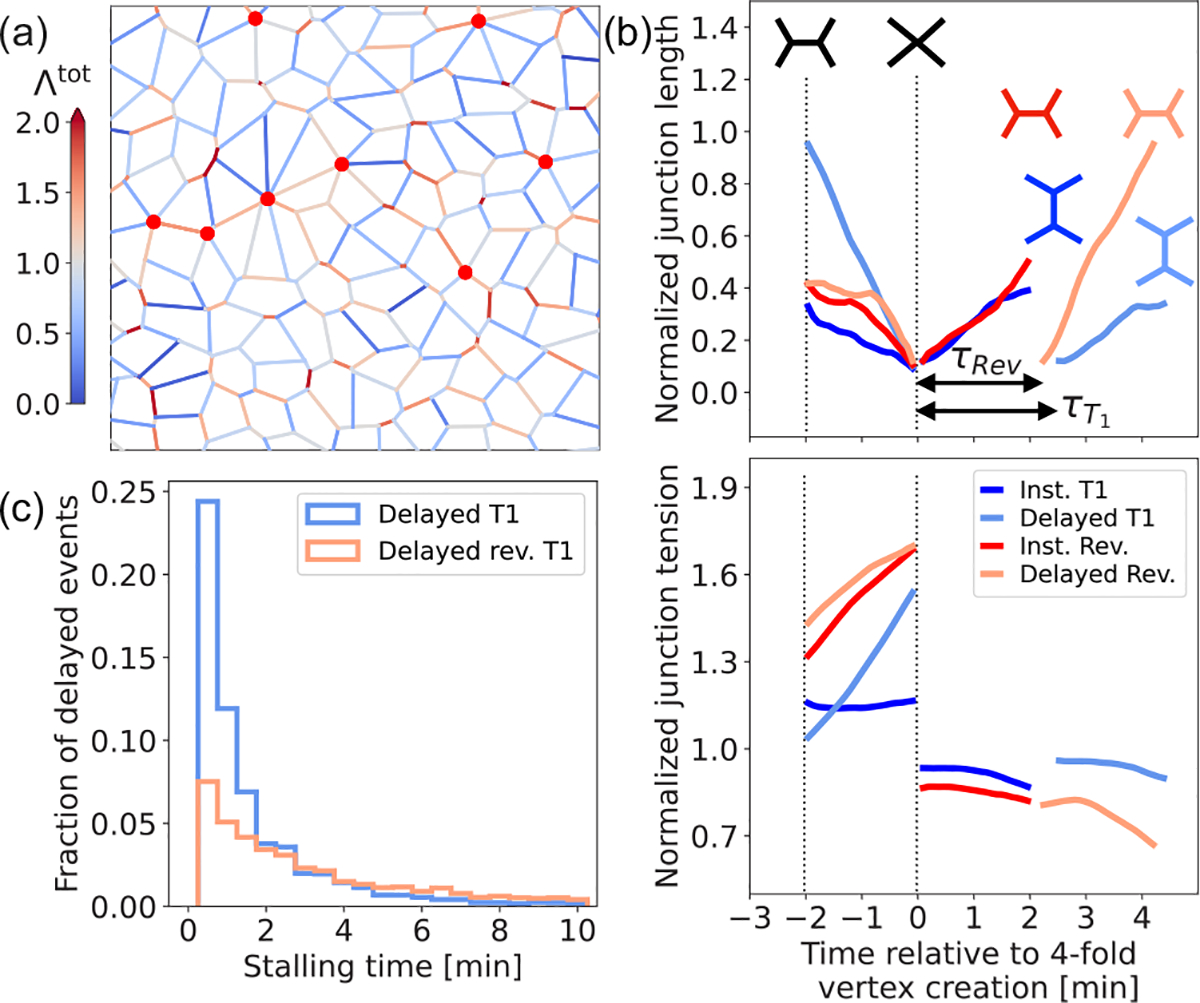
Delayed T1 transitions and intercalation dynamics in remodeling tissues. (a) Representative section of a simulated epithelial tissue with tension remodeling ($k_{C}/k_{L}=0.2$ and $k_{E}/k_{L}=0.17$) at t~6 h. Red circles represent fourfold vertices and colored cell edges represent the total tension, in units of $\Lambda_{0}$. (b) Normalized junction length (top) and tension (bottom), as a function of time relative to the fourfold vertex creation, for instantaneous and delayed T1 events. (c) Histogram of the stalling time for delayed irreversible T1 (blue) and delayed reversible T1 (red) events.

To characterize the role of tension remodeling on T1 transitions, we first simulate a disordered tissue comprising approximately 500 cells in a box with periodic boundary conditions, as in [44]. In simulations, we nondimensionalize force scales by $K{\left(A_{\alpha }^{0}\right)}^{3/2}$, length scales by $\sqrt{A_{\alpha }^{0}}$, and timescales by $\mu /KA_{\alpha }^{0}$, setting $K=1,\left\langle A_{\alpha }^{0}\right\rangle =1$, and μ=0.2 (approximately 28 s), where ⟨⋯⟩ represents the population average. The initial state of the simulations is characterized by having zero initial junction strain $\left(l_{ij}=l_{ij}^{0}\right),\left\langle l_{ij}^{0}\right\rangle \sim 0.62$, and $\left\langle \Lambda_{ij}\right\rangle =\Lambda_{0}=0.1$, which is also the mean value for the tension of a newly created junction (see the Supplemental Material [43], Fig. S1). We let the tissue evolve from an energy relaxed state with chosen values of active contractility $\Gamma_{a}$, active fluctuations of amplitude σ, threshold strain $\varepsilon_{c}=0.1$, and strain relaxation rate $k_{L}$, with different values for the tension remodeling rates $k_{E}$ and $k_{C}$. A representative tissue snapshot is shown in Fig. 2(a), for a particular simulation using $k_{C}/k_{L}=0.2$ and $k_{E}/k_{L}=0.17$, which displays multiple (transiently stable) fourfold vertices (red circles) representing stalled T1 transitions (Movie 1).

Four different types of dynamics are observed during T1 processes [see Fig. 2(b) and Movie 1]: instantaneous irreversible T1 events, delayed irreversible T1 events with a stalled fourfold vertex, instantaneous reversible events, and delayed reversible T1 events. In all these cases, tension increases during contraction prior to fourfold vertex formation, as a consequence of tension remodeling. Subsequently, tension decreases via remodeling after the fourfold vertex is resolved into an extending junction [Fig. 2(b)]. Tension remodeling can decrease the local tensions in stretched shoulder junctions, promoting fourfold stabilization. Specifically, a stalled fourfold vertex arises when $f_{\text{fourfold}}=\left(f_{i}-f_{j}\right)\cdot {\overset{ˆ}{r}}_{ij}<2\Lambda_{\text{birth}}$, where ${\overset{ˆ}{r}}_{ij}=\left(r_{i}-r_{j}\right)/\left|r_{i}-r_{j}\right|$ and $f_{i}$ and $f_{j}$ are the forces acting on the two tricelullar vertices i and j created in the attempt of fourfold vertex resolution. These forces arise from tensions in the shoulder junctions as well as pressures in the neighboring cells resisting changes in the cell area. When the local tension increases due to strain-driven remodeling or contractility, vertex stability is lost, resulting in a delayed T1 or a delayed reversible event [Fig. 2(b)]. Figure 2(c) shows the distribution of stalling times for both reversible and irreversible T1 events, suggesting that some fourfold vertices can be resolved nearly instantaneously, while others can remain stalled for longer periods. Without tension remodeling $\left(k_{E}=k_{C}=0\right)$, we recover the standard vertex model where T1 transitions occur instantaneously and fourfold vertices are unstable (Movie 2). For negative values of the tension remodeling rates, we obtain a model of positive feedback between tension and strain, where T1 transitions are observed to occur instantaneously (see the Supplemental Material [43], Fig. S3).

**FIG. 3.**
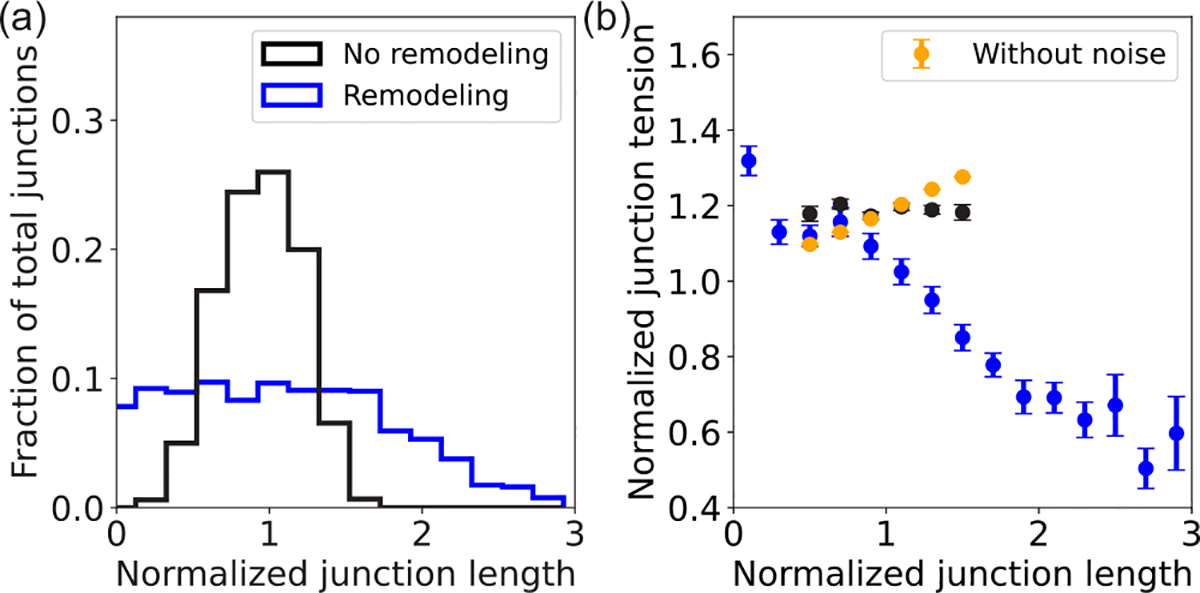
Tension remodeling promotes asymmetric length distribution. (a) Histogram of junction length at steady state, for a tissue with remodeling (blue, $k_{C}/k_{L}=0.2$ and $k_{E}/k_{L}=0.17$) and without remodeling (black, $k_{C}=k_{E}=0$). (b) Correlation between total junction tension (normalized) and junction length (normalized) in tissues with (blue) and without (black) junction remodeling. The orange data points show the positive correlation between deterministic tension (i.e., junction tension without the fluctuating part) and junction length, in the absence of junctional tension remodeling. Error bars represent ±1 standard error of mean.

The dynamics of the model tissue with tension remodeling, as characterized in Fig. 2 (Movie 1), settles into a fluctuating steady state with an asymmetric distribution of junction length [Fig. 3(a)], as observed in mature *Drosophila* epithelium [17]. Furthermore, a negative correlation is observed between junction length and tension in the fluctuating steady state [Fig. 3(b)], analogous to the negative correlation between junction length and myosin intensity seen experimentally [17,45]. By contrast, without tension remodeling $\left(k_{E}=k_{C}=0\right)$, the junction length distribution is symmetric [Fig. 3(a)]. In this case, junction length is positively correlated with the deterministic part of the tension $\left(\Lambda_{0}+\Gamma_{a}l_{ij}\right)$ [orange dots in Fig. 3(b)]. However, this positive correlation is lost when we consider the total junction tension, including the fluctuating part, since the amplitude of the tension fluctuations (σ) is comparable to $\left\langle \Gamma_{a}l_{ij}\right\rangle$ [black dots in Fig. 3(b)].

### B. Stability of fourfold vertices

During a T1 transition, the fourfold vertex can be transiently stable if the tension in the extending shoulder junctions is low compared to the tension in the newly created junction. This can be achieved via tension remodeling in the extending shoulder junctions, controlled by the rate $k_{E}$. To understand how $k_{E}$ affects vertex stability, we study an effective meanfield model consisting of symmetric cell junctions embedded in an effective elastic medium (see the Supplemental Material [43], Fig. S4). We activate contraction in chosen junctions by increasing $\Gamma_{a}$. During this process, the contracting (extending) junctions increase (decrease) their tension at a rate $k_{C}\left(k_{E}\right)$. We find that if $\beta k_{E}>k_{C}$, the global tissue tension decreases, promoting mechanical stability of the fourfold vertex, where β is the ratio of the total length gained by the extending junctions to the total length lost by the contracting junctions.

**FIG. 4.**
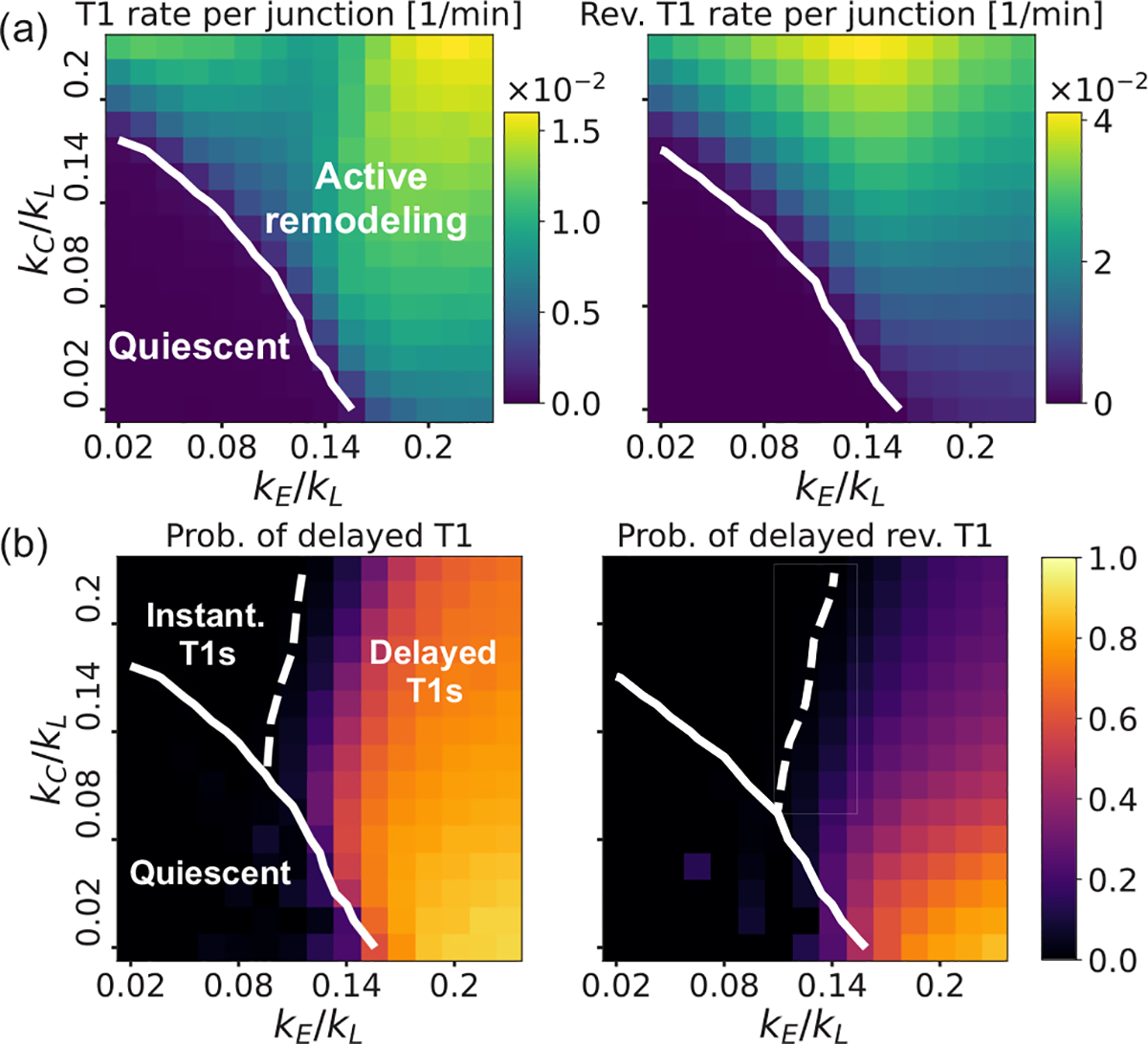
Junction tension remodeling regulates T1 transition rates. (a) Rates of T1 (left) and reversible (right) transitions for different values of $k_{E}/k_{L}$ and $k_{C}/k_{L}$. Solid lines represent 10^−3^ T1 events per junction per minute. (b) Probability of delayed irreversible T1 transitions (left) and reversible T1 events (right) for different values of $k_{E}/k_{L}$ and $k_{C}/k_{L}$. Dashed line represents 1% probability.

To further investigate the role of tension remodeling on T1 transitions, we perform numerical simulations using different values of the tension remodeling rates $k_{C}/k_{L}$ and $k_{E}/k_{L}$ in the range [0.02,0.23] (Fig. 3). From fits to experimental data on single-junction deformations, it is determined that $k_{C}/k_{L}\approx 0.14$ [28,31] and $k_{E}/k_{L}\approx 0.12$ [32]. We find that the tension remodeling rates control the rate of T1 transitions as well as the probability of delayed T1 transitions. For very small values of $k_{E}$ and $k_{C}$, the tissue is in a quiescent state, where T1 events are scarce (fewer than 10^−4^ per junction per minute) and occur instantaneously [Fig. 3(a)]. Due to the lack of appreciable tension remodeling in the quiescent state, tension in the shoulders of an intercalating junction is approximately $\Lambda_{0}$, with negligible resistive pressure in the surrounding cells since $A_{\alpha }\sim A_{\alpha }^{0}$. As a result, $f_{\text{fourfold}}$ remains larger than $2\Lambda_{\text{birth}}$, making the fourfold vertex unstable. For larger values of $k_{C}$ and $k_{E}$, irreversible and reversible T1 events are mainly driven by tension remodeling, inducing wider pressure distributions (Fig. S5 [43]) and higher rates of T1 events [Fig. 4(a)]. In this parameter regime, $f_{\text{fourfold}}$ depends on both tensions and pressures in the surrounding cells. Since the tension remodeling dynamics is fast compared to pressure relaxation, pressurelike forces make $f_{\text{fourfold}}$ larger in the original direction of contraction, turning the reversible T1 events more probable. In the presence of tension remodeling, T1 events either occur instantaneously or are delayed, with probabilities given in Fig. 4(b). The probabilities of delayed events, and hence the presence of stalled fourfold vertices, depend strongly on $k_{E}$, as predicted analytically. Fourfold stability increases for large $k_{E}$ and small $k_{C}$, reaching stalling times of 5 min on average (Fig. S6 [43]), consistent with experimental data [13].

It is important to note that the phase diagrams for the T1 rate per junction, probability of delayed T1 transitions (Fig. 4), and the probability distributions of T1 stalling times remain qualitatively the same for different choices of strain threshold parameter $\varepsilon_{c}$, length of newly created junctions $l_{\text{birth}}$, and tension resetting rules upon T1 transitions (see the Supplemental Material [43], Figs. S7–S12).

### C. Mechanical memory dissipation promotes T1 transitions

**FIG. 5.**
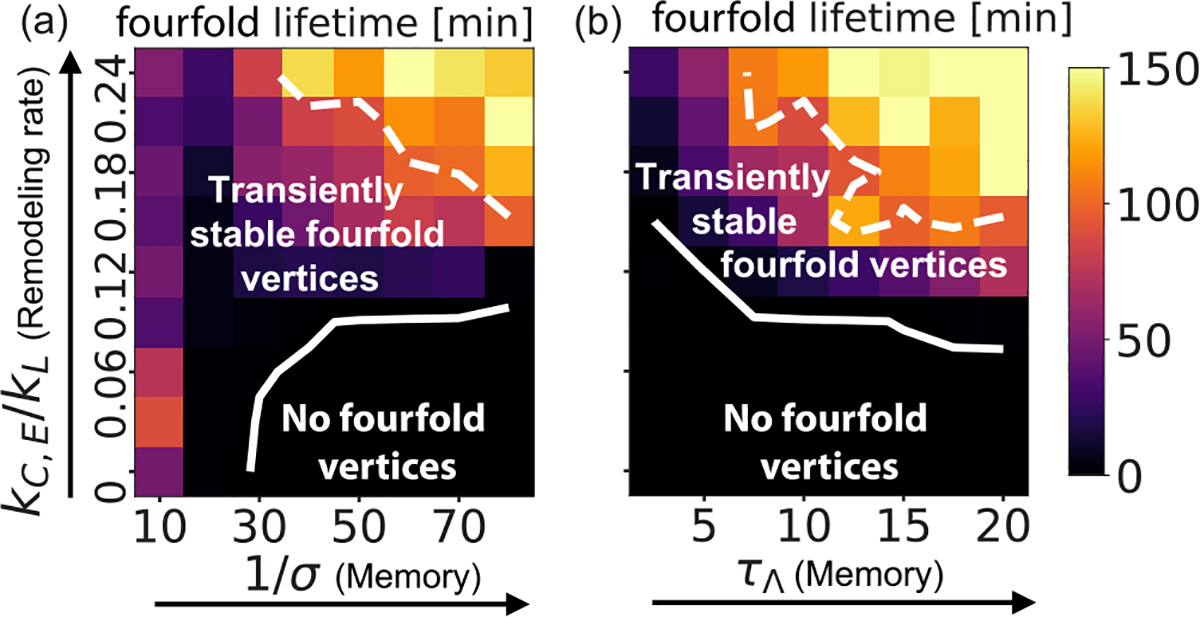
Mechanical memory dissipation promotes T1 transitions. The maximum lifetime of fourfold vertices is plotted as a function of tension remodeling rate $k_{C}/k_{L}$ (with $k_{C}=k_{E}$) and the regulators of mechanical memory: (a) inverse of noise amplitude 1/σ (with $\tau_{\Lambda }=10$) and (b) tension relaxation timescale $\tau_{\Lambda }$ (with 1/σ=50). The dashed contour represents a 100-min lifetime. Below the solid line, there are fewer than 10^−3^ fourfold vertices resolved per junction per minute.

**FIG. 6.**
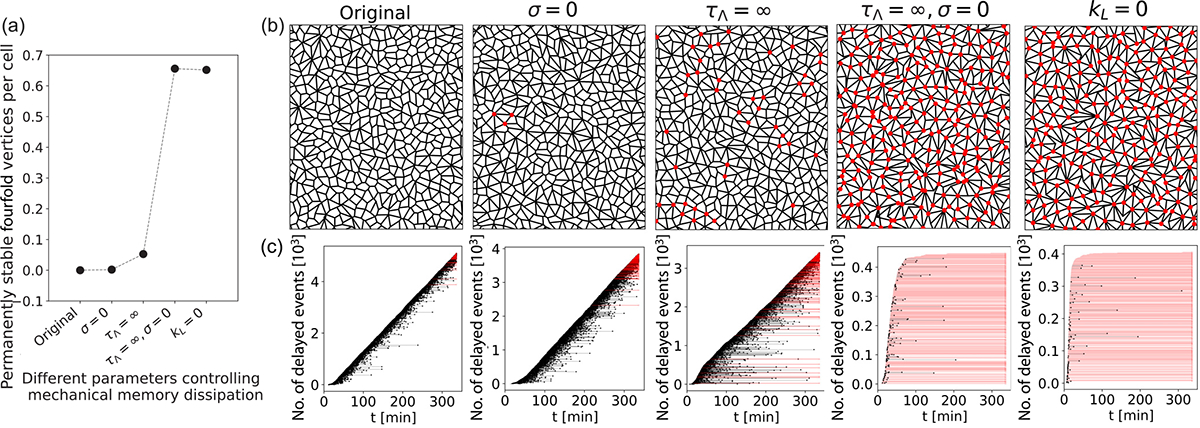
Transient stability of fourfold vertices relies on mechanical memory dissipation. (a) Permanently stable fourfold vertices per cell, for an active tissue with $k_{C}=0.1$ and $k_{E}=0.2$ and for different parameters controlling mechanical memory dissipation, in the following order (from left to right): (i) original simulation with all modes of memory dissipation, with finite $\sigma ,k_{L}\neq 0$, and $\tau_{\Lambda }^{-1}\neq 0$; (ii) no tension fluctuations, with $\sigma =0,k_{L}\neq 0$, and $\tau_{\Lambda }^{-1}\neq 0$; (iii) no tension relaxation, with finite $\sigma ,k_{L}\neq 0$, and $\tau_{\Lambda }^{-1}=0$; (iv) no tension fluctuations and tension relaxation, with $\sigma =0,k_{L}\neq 0$, and $\tau_{\Lambda }^{-1}=0$; and (v) no strain relaxation, with finite $\sigma ,k_{L}=0$, and $\tau_{\Lambda }^{-1}\neq 0$. (b) Tissue configurations showing the steady-state morphology (at approximately 350 min), where red solid circles represent fourfold vertices that have been stable for more than 100 min by the end of each simulation. Simulations with strain relaxation consider $k_{L}=1$. (c) Number of delayed T1 events vs time, corresponding to the simulated tissues shown in (b). Each horizontal line represents the creation of a fourfold vertex. Black lines represent fourfold vertices that are resolved through the simulation (at the time highlighted by a black dot), over a timescale longer than 6 s. Red lines that finish in an empty edge-colored red circle represent fourfold vertices that are not resolved during the simulation.

Our study so far demonstrates that tension remodeling rates control the probability of T1 events as well as the stability of fourfold vertices. Since higher-order vertices appear transiently in living tissues [11–13,17], we wondered what tissue properties would regulate the lifetime of fourfold vertices. To that end, we found that T1 stalling time increases with both the inverse of the noise magnitude 1/σ and the timescale of stress relaxation $\tau_{\Lambda }$ (see Figs. 5, S13, and S14 [43]). On the contrary, the rate of T1 events increases with σ [3,17,46] and decreases with $\tau_{\Lambda }$ (Figs. S13 and S14 [43]). For low or no noise (σ=0), tension fluctuations are diminished with fourfold vertices being present for more than 100 min [see Figs. 5(a) and 6(c) and Movie 3]. Interestingly, for very high σ (limit of no mechanical memory), tissues can reach certain geometrical configurations that allow the existence of stalled fourfold vertices even in the absence of tension remodeling [Fig. 5(a)]. However, in such instances the stalling time cannot be dynamically controlled. For intermediate levels of noise, active tension remodeling induces fourfold vertex formation with controllable lifetime. Additionally, without tension relaxation $\left(\tau_{\Lambda }=\infty \right)$ or strain relaxation $\left(k_{L}=0\right)$, tissues develop permanently stable fourfold vertices (Fig. 6). In particular, for the cases $k_{L}=0$ and $\left\{\tau_{\Lambda }=\infty ,\sigma =0\right\}$, the system quickly gets stuck in geometrical configurations with a high density of stable fourfold vertices. Our model also leads to mechanical memory dissipation via tension resetting during a fourfold vertex resolution into threefold vertices. We find that a persistent-tension rule during a T1 transition also leads to permanently stable fourfold vertices (see Fig. S15 [43]), as seen experimentally in *Drosophila* (pupal wing) lacking the tumor suppressor PTEN [45].

Thus, transiently stable fourfold vertices, defined by having stalling times shorter than 100 min, require two fundamental ingredients (Fig. 5): (i) a negative feedback between junctional tension and strain (tension decreases with increasing strain) and (ii) mechanical memory dissipation via strain relaxation $\left(k_{L}\neq 0\right)$, tension relaxation (finite $\tau_{\Lambda }$), and noiseinduced fluctuations (intermediate 1/σ). While there are other recent models with tension-strain feedback [29,30,41,47], those do not concurrently satisfy the above two specific criteria for tension remodeling and mechanical memory dissipation and therefore cannot capture transiently stable fourfold vertices (see Sec. IV).

### D. Tension remodeling rates control tissue material properties

**FIG. 7.**
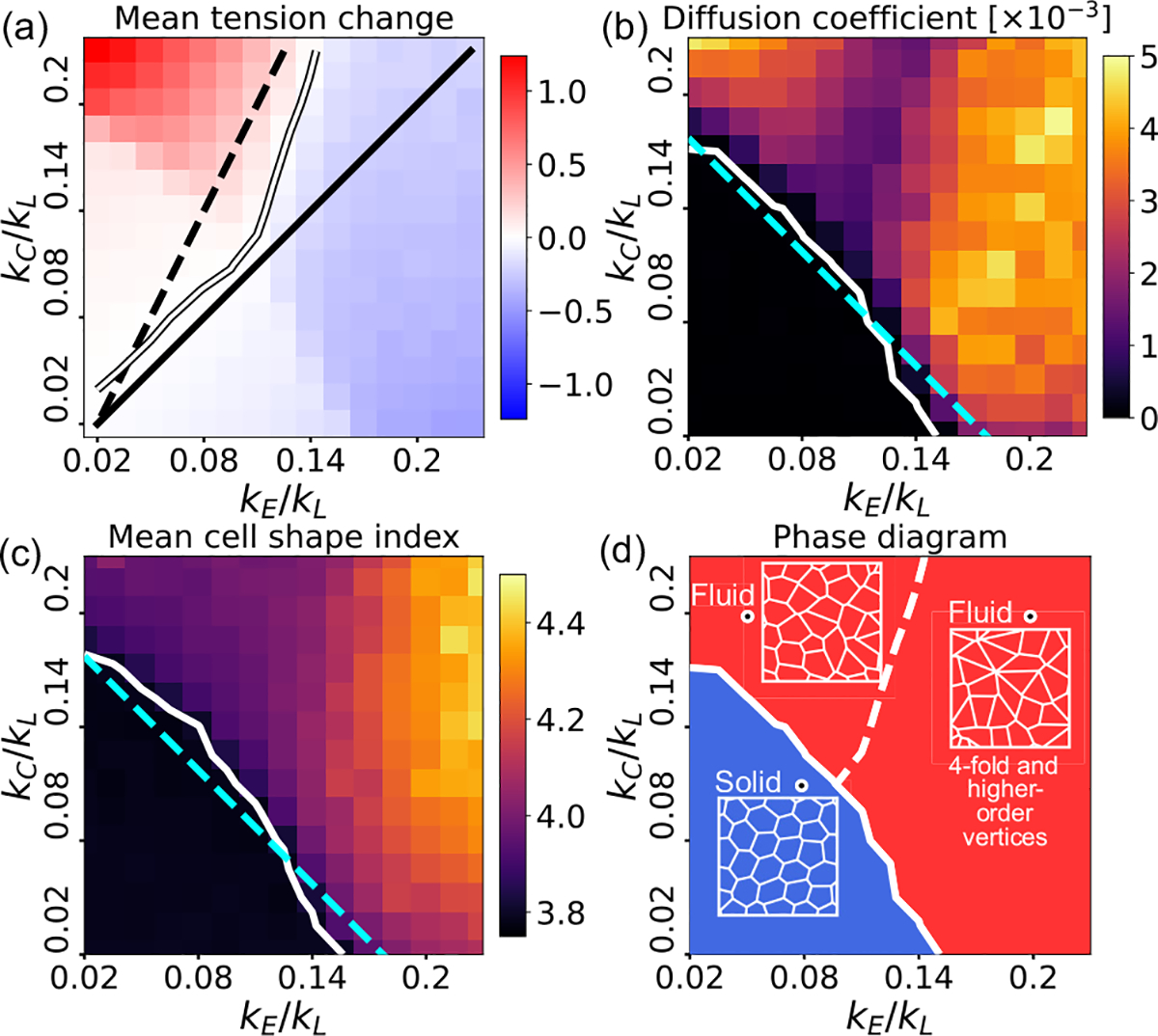
Emergent tissue mechanics from cell junction remodeling. (a) Colormap of mean tension change (in units of $\Lambda_{0}$) at steady state of the remodeled tissue, as a function of $k_{E}/k_{L}$ and $k_{C}/k_{L}$. Black (white) curves correspond to numerical results obtained with the effective medium models (simulations), representing no change in tension. The black dashed curve shows $2k_{E}=k_{C}$ and the black solid curve $k_{E}=k_{C}$. (b) Diffusion coefficient D as a function of $k_{E}/k_{L}$ and $k_{C}/k_{L}$. The white curve represents $D=10^{-4}\left[{\left\langle A_{0}^{\alpha }\right\rangle }^{2}/\text{min}\right]$. (c) Mean cell shape index q as a function of the tension remodeling rates. The white curve represents q=3.81. The cyan dashed line in (b) and (c) represents the prediction of the mean-field model, with fitted effective medium stiffness k=0.09. (d) Phase diagram showing transitions between solid (blue) and fluid (red) states of the tissue, with the white solid curve representing the phase boundary. Below the white dashed curve in the fluid phase, stable fourfold and higher-order vertices are prevalent.

Tension remodeling rates not only control the kinetics of T1 transitions and tissue topology, but also regulate tissue material properties. To characterize mechanical properties at the tissue level, we first examine the effects of junction remodeling on average tissue tension, since low tension is associated with fluidlike tissues whereas high tension promotes solidity [3,48]. To this end, we compute the mean change in tissue tension from an initial steady state, as a function of the tension remodeling rates $k_{E}$ and $k_{C}$ [Fig. 7(a)]. Here the initial state is chosen as the steady state of the tissue with a constant mean junctional tension $\langle \Lambda \rangle =\Lambda_{0}$ and zero junctional strain. Therefore, any changes in mean tissue tension would reflect the effects of junction tension remodeling, resulting in junction length variations.

We find that the mean tension change is negative in the parameter space with nonzero probabilities of delayed T1 transitions [Fig. 4(b)], suggesting a loss of tissue rigidity. The white solid line in Fig. 7(a) represents the phase boundary obtained from simulations, where the mean tissue tension does not change. For small $\left(k_{E},k_{C}\right)$, the phase boundary follows the line $k_{E}=k_{C}$ (black solid), as predicted by the mean-field model in a system conserving the total junction length (see the Supplemental Material [43]). For large $\left(k_{E},k_{C}\right)$, the phase boundary increases in slope, as predicted in a system increasing its total junction length. In a two-dimensional mean-field model, consisting of five symmetric cell junctions subject to an increase in total junction length (see the Supplemental Material [43]), the predicted phase boundary is $2k_{E}=k_{C}$ (black dashed line). To directly test the role of tension remodeling on tissue mechanical properties, we perform finite shear simulations (Fig. S16 [43]). These simulations reveal that tissues with high-tension remodeling, exhibiting transiently stable fourfold vertices and a negative mean tension change, are associated with an enhanced rate of energy and stress release. From the measurements of changes in tissue tension, we find that tissues with smaller values of $k_{E}$ and $k_{C}$ maintain a constant mean tension [Fig. 7(a)], with very low rates of neighbor exchange [Fig. 4(a)], characteristic of an arrested state. To quantify cell movement, we measure the mean-square displacements of the cell centers (Supplemental Material [43]) to compute the average diffusivity D of cells [Fig. 7(b)]. We find that cells with smaller values of $\left(k_{E},k_{C}\right)$ do not diffuse significantly ($D<10^{-4}$), representing solidlike tissues with mostly hexagonal cell shapes. Diffusivity increases with $k_{E}$ and $k_{C}$ such that the tissue is liquidlike when $\left(k_{E}+k_{C}\right)/k_{L}$ is larger than a critical value [Fig. 7(b)]. Interestingly, tissues possessing higher-order vertices are fluidlike with a high cell diffusivity [Fig. 7(b)]

In vertex models describing isotropic tissues, fluidity is related to the observed cell shape index q [4,48,49], defined as the mean ratio between the perimeter and the square root of the cell area. A fluid-solid phase transition occurs at q=3.81 such that the tissue is solidlike for q<3.81. The rigidity transition is related to the mechanical stability of junctions, which occurs in the mean-field theory when $\left(k_{C}+k_{E}\right)/2k_{L}$ is smaller than the effective medium stiffness k [dashed line in Figs. 7(b) and 7(c); see the Supplemental Material [43]]. From our simulations we obtain excellent agreement between the contours $D=10^{-4}$ and q=3.81 [white curves in Figs. 7(b) and 7(c)]. Our theory thus relates the fluidity of confluent tissues and their emergent topology to the rates of tension remodeling $k_{C}$ and $k_{E}$ (Fig. 7). In particular, we find that fourfold and higher-order vertices can become stable in fluid tissues if $\beta k_{E}>k_{C}(\beta >1)$ such that asymmetric tension remodeling reduces mean tissue tension.

### E. Tension remodeling regulates the geometry of cell packing

**FIG. 8.**
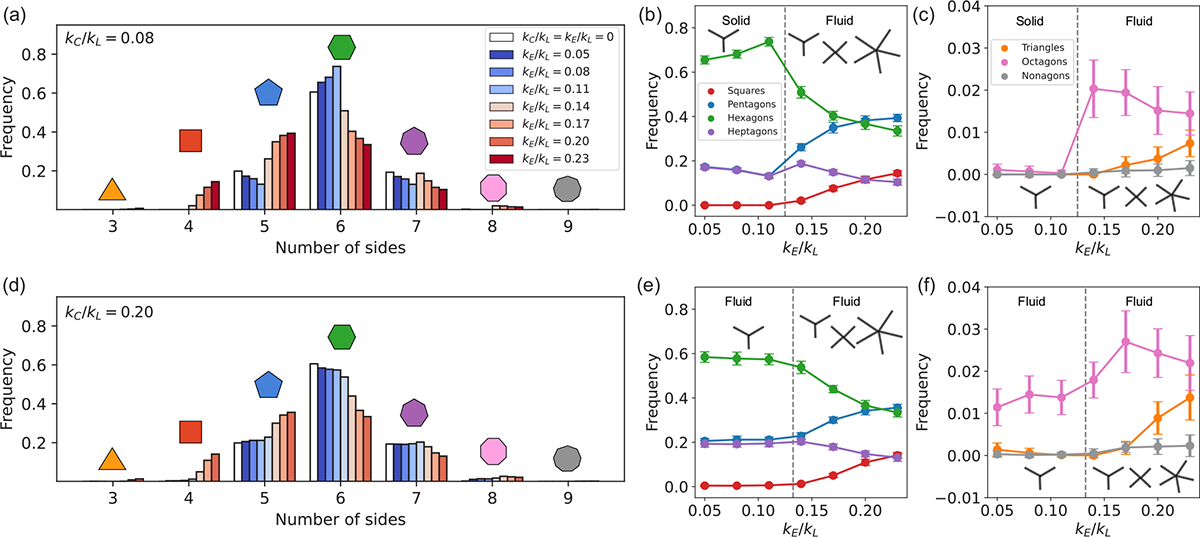
Tension remodeling influences the geometry of cell packing. The mean frequency of polygon sidedness in tissues with (a)–(c) $k_{C}/k_{L}=0.08$ and (d)–(f) $k_{C}/k_{L}=0.20$ is plotted as a function of $k_{E}/k_{L}$, considering 20 random initial seeds for each simulation. White bars in (a) and (d) show the distribution of polygon sidedness in tissues without tension remodeling ($k_{E}=k_{C}=0$). Error bars in (b), (c), (e), and (f) represent ±1 standard deviation.

In addition to controlling the frequency and timescale of T1 transitions, and tissue material properties, tension remodeling also influences the geometry of cell packing in epithelia. To characterize the cell packing geometries, we measure the fraction of cells in different polygon classes, characterized by their number of sides. Figure 8 shows the distribution of the number of polygon sides for different values of $\left(k_{C}/k_{L},k_{E}/k_{L}\right)$, along with the polygon sidedness for tissues with no tension remodeling (white bars). In tissues without tension remodeling, only pentagons, hexagons, and heptagons are observed in the ground state of the vertex model. We find that increasing $k_{E}$ in a solid tissue increases the number of hexagonal cell shapes while decreasing the relative numbers of pentagons and heptagons. The behavior is similar to what was obtained in Ref. [17], when increasing the mean line tension in the cell edges. On the other hand, when increasing $k_{E}$ in a fluid tissue, the numbers of hexagons and heptagons decrease, while pentagons increase in number. In fluid tissues, triangles, squares, octagonal, and nonagonal cell shapes are also observed as $k_{E}$ is increased.

Experimental data demonstrate the presence of diverse polygonal cell shapes, ranging from triangles to nonagons, in various tissues such as the larval wing disc of *Drosophila* [21,50], tail epidermis of *Xenopus* [50], and the epidermis of *Hydra* [50]. However, the origin of these irregular cell packing geometries, whether arising from cell divisions, anisotropic forces within the tissue, active tension remodeling, or a combination thereof, remains inconclusive. Here we establish that tension remodeling alone is sufficient to induce irregularities in cell packing. Future investigations that integrate cell divisions with tension remodeling and anisotropy will provide quantitative insights into the relative contributions of each of these factors in governing cell packing geometry in disordered epithelia.

## IV. DISCUSSION

One common assumption in existing cell-based models of epithelial tissues is that epithelia resemble foamlike networks, consisting of bicellular junctions that connect tricellular vertices [14,15,21]. More complex structures, such as rosettes, where four or more junctions meet, are widely observed *in vivo* [11–13,17] but are not stable structures in existing vertex models. In this paper we provided the first theoretical model for the spontaneous emergence of stable higher-order vertices and elucidated the underlying physical principles that regulate their assembly and lifetime. In particular, we identified two general physical principles that are necessary and sufficient for the formation and transient stability of higher-order vertices: (i) strain-dependent tension remodeling (specifically, a negative feedback between tension and strain) and (ii) mechanical memory dissipation. First, we showed that the ability of cellular junctions to actively decrease tension under extension and increase tension under contraction promotes the formation of fourfold vertices that are precursors to T1 transitions. The model for strain-dependent tension remodeling was derived from recent studies on single-junction mechanics [28,31]. Second, the relaxation of mechanical strain, tension, and noise-induced fluctuations enables the dissipation of mechanical memory over time, which is necessary for the timely resolution of fourfold vertices. Resolution of fourfold vertices can occur instantaneously or noninstantaneously, resulting in reversible or irreversible T1 transitions. In particular, T1 resolution or stalling time increases with both 1/σ and $\tau_{\Lambda }$. For very small values of tension remodeling rates, we found that the classical vertex model results are recovered, where fourfold vertices are unstable and resolved through T1 events.

Our modified version of the vertex model treats each junction as an independent entity, in contrast to the classical vertex model with a perimeter-dependent contractility term. This distinction is particularly significant because the cell perimeter-dependent model introduces nonlocal tensions in newly formed junctions following a T1 transition, which depends on the shapes of the two adjacent cells. While there is no experimental evidence supporting this nonlocal tension term, its inclusion in the model can result in stabilization of fourfold vertices under specific initial conditions of cell shapes, assuming they follow similar force-dependent rules for the formation and resolution of fourfold vertices, as in our model. However, the existence of these fourfold vertices is reliant on the choice of initial geometrical conditions.

In recent years, many studies have delved into the profound impact of mechanical feedback on epithelial tissue dynamics [29,30,41,47]. These include studies that considered a positive-feedback between tension and strain [30,41,47,51,52] or a negative-feedback between tension and strain akin to our model [28,29,32,37]. However, none of these models fulfill the combined requirements of negative-feedback-based tension remodeling and memory dissipation, thus falling short in capturing the transient stability of higher-order vertices within epithelial tissues. In the active tension network model introduced by Noll *et al*. [47] and later developed by Gustafson *et al*. [30], tissue dynamics is governed by tension remodeling without elastic restoring elastic forces. Opposite to our work, the authors considered that tension increased in elongated junctions and decreased in contracted junctions, which was necessary to ensure mechanical stability in the absence of elastic restoring forces. Under such rules, fourfold vertices cannot be stabilized as the net pulling force in the extending shoulder junctions would always exceed the force between two proximal tricellular vertices. On the other hand, Krajnc *et al*. [29] considered physical rules for active tension remodeling similar to those of Staddon *et al*. [28], but did not include mechanisms for strain relaxation that we found to be necessary to avoid permanently stable fourfold vertices (see Fig. 6). A recent study by Sknepnek *et al*. [41] implemented specific rules for mechanosensitive myosin dynamics that regulated cell junction tension. In contrast to our model, Sknepnek *et al*. found that the tension in the shoulder junctions of a contracting junction increases, which promotes the instability of the fourfold vertices as argued by us. Second, Sknepnek *et al*. did not consider relaxation and remodeling of passive tensions in the vertex model. In the absence of total tension relaxation, permanently stable fourfold vertices would arise (see Fig. 6).

We showed that by tuning the values of the tension remodeling rates $k_{C}$ and $k_{E}$, the tissue can be driven through two distinct phase transitions. First, the model predicts a rigidity crossover if $\left(k_{C}+k_{E}\right)/k_{L}$ is smaller than a critical value, which corresponds to a mean observed cell shape index of 3.81. Below the rigidity threshold, T1 events are scarce and tissues are highly ordered, with a high fraction of hexagonal cells, followed by smaller fractions of pentagons and heptagons. Increasing the tension remodeling rates above the rigidity threshold leads to an increase in T1 events, with a wider distribution of polygon sides (from triangles to nonagons) and an asymmetric distribution of junction length, as seen in experimental data [17]. Our model thus belongs to a broader class of vertex models with universal rigidity features as suggested in Ref. [49]. Second, our model predicts a transition in tissue topology from unstable to stable fourfold vertices in the fluid phase. In this phase, mechanical stability is reached with a lower value of mean tension, and there is asymmetry in the rates of tension remodeling in response to junction contraction and extension. Stable fourfold vertices can also lead to the formation of even higher-order vertices, as shown in Movie 4, where we allowed up to fivefold vertices. It is important to recall that our model assumes an isotropic tissue. In anisotropic tissues cell shape may not be a direct proxy for fluidity [53]. This could explain the presence of stable fourfold vertices in tissues with isotropic cell shapes (low shape index) as the *Drosophila pupal* wing [10].

Previous studies have enforced the creation of fourfold and higher-order vertices in canonical vertex models [23] and imposed *ad hoc* rules for the stalling of T1 events [13,24,25]. For instance, Finegan *et al*. [13] implemented probabilities for successful T1 resolution and imposed the no resolution of rosettes, while Das *et al*. [24] and Erdemeci-Tandogan and Manning [25] introduced clocks for T1 transitions. In our model, the stability of higher-order vertices is naturally linked to the mechanical state of the tissue; in particular, they arise in low-tension tissues. Interestingly, an increase in the mean tension in this model does not imply a more solidlike tissue. Instead, high-tension systems are obtained in tissues with high rates of instantaneous T1 events (Supplemental Material [43]), inducing cellular motion through these topological rearrangements and increasing diffusion, making the tissue more fluidlike. It has been previously reported that the presence of higher-order vertices leads to rigidification of tissues [23]. While we did not directly evaluate shear modulus of the tissue, we found that the presence of stalled fourfold vertices reduces the rate of instantaneous cell neighbor exchanges (Fig. S17 [43]). In addition, the mechanical stability of fourfold vertices demands a liquidlike tissue with low overall tension. This implies there are many T1 events occurring in the presence of stable fourfold vertices, as observed during *Drosophila* axis elongation [13].

## Supplementary Material

Supplemental Material

## Figures and Tables

**Movie 1:** 

**Movie 2:** 

**Movie 3:** 

**Movie 4:** 
