# Supplementary material for: Tension Remodeling Regulates Topological Transitions in Epithelial Tissues
Source: PRX Life. Author manuscript; Available in PMC 2024 Oct 24. (PMC11500814; doi:10.1103/prxlife.1.023006)
Supplement: Supplemental Material [file NIHMS1984388-supplement-Supplemental_Material.pdf]

Supplemental Material

Tension remodeling regulates topological transitions in epithelial tissues

Fernanda Pérez-Verdugo[1] and Shiladitya Banerjee[1]

[1] Department of Physics, Carnegie Mellon University, Pittsburgh, PA 15213, USA

1 Simulation Methods

1.1 Initial configuration

We created a disordered tissue composed of 494 cells in a box of dimensions $L_x \times L_y$ (Table 1), with periodic boundary conditions. The disordered tissue was built via Voronoi tessellation, where the positions of the cell centers were generated by a Monte Carlo simulation of hard disks, with an area fraction equal to 0.71 [1]. On this disordered tissue comprising variable-sided polygons, we obtained the energy relaxed state by evolving the system using the standard vertex model Hamiltonian:

$$E = \frac{1}{2}K\sum_{\alpha}\left(A_\alpha - A_\alpha^0\right)^2 + \frac{1}{2}K_P\sum_{\alpha}\left(P_\alpha - P_\alpha^0\right)^2, \tag{1}$$

where P_α and $P_\alpha^0 = P_0$ define the cellular perimeters and their target values, respectively. We used $K = 1, K_P = 0.2, P_0 = 3.5$, and A_α^0 was drawn from a normal distribution with unit mean and standard deviation 0.1. During the initial energy relaxation process, we allowed T1 rearrangements when cellular edges became smaller than a length threshold $l_{T_1} = 0.05$ and if the topology change decreased the overall tissue energy. This procedure ensured that the previous relaxed tissue configuration is in a solid state [2] containing tricellular vertices only. Using the configuration of the resulting tissue, we initiated a second round of energy relaxation using the hamiltonian defined in Eq. (1) of the main text, with active terms set to zero. During this process, we preserved the values of the target areas, and assumed that the initial rest length l_{ij}^0 is equal to the initial junction length l_{ij}. Further, cell junctions were assigned tension values Λ_{ij}, drawn from a uniform distribution with mean 0.1 and standard deviation 0.01. The parameter values are given in Table 1. We let this tissue relax its energy by setting $k_E = k_C = 0$, $\sigma = 0$, $\Gamma_a = 0$. As a result, the tensions Λ_{ij} did not change, while l_{ij}^0 and l_{ij} reach approximately normal distributions with mean 0.62 (side of a regular hexagon with unit area), and standard deviation ~ 0.2. The relaxed state, defining the initial configuration of our simulations, represents a solid-like tissue [3] with tricellular vertices only, and polygonal shapes from squares to octagons (Fig. S1). Note that this relaxed state of the tissue could have been obtained only using the second relaxation step. However, our two-step method minimizes the noise in the system arising due to the creation and resolution of 4-fold vertices, making the relaxation process much faster.

Parameter	Symbol	Value
Area elastic modulus	K	1
Mean preferred area	$\langle A_\alpha^0 \rangle$	1
Friction coefficient	μ	0.2 (28 s)
Mean initial tension	Λ_0	0.1
Tension in newly created junction	Λ_{birth}	0.1
Length of a newly created junction	l_{birth}	1.5 l_{T_1}
Active contractility	Γ_a	0.03
Noise amplitude	σ	0.02
Simulation box length	L_x	~ 20
Simulation box width	L_y	~ 24
Strain relaxation rate	k_L	1
Tension remodeling rate under contraction	k_C	$\in [0.02, 0.23]$
Tension remodeling rate under extension	k_E	$\in [0.02, 0.23]$
Critical strain threshold	ε_c	0.1
Length threshold for attempting T1 transitions	l_{T_1}	0.05
Time between attempts to resolve 4-fold vertices	τ_{test}	0.04
Tension relaxation timescale	τ_Λ	10
Relaxation timescale for tension fluctuations	τ	0.4
Integration time step	Δt	0.004

Table 1: Model parameters.

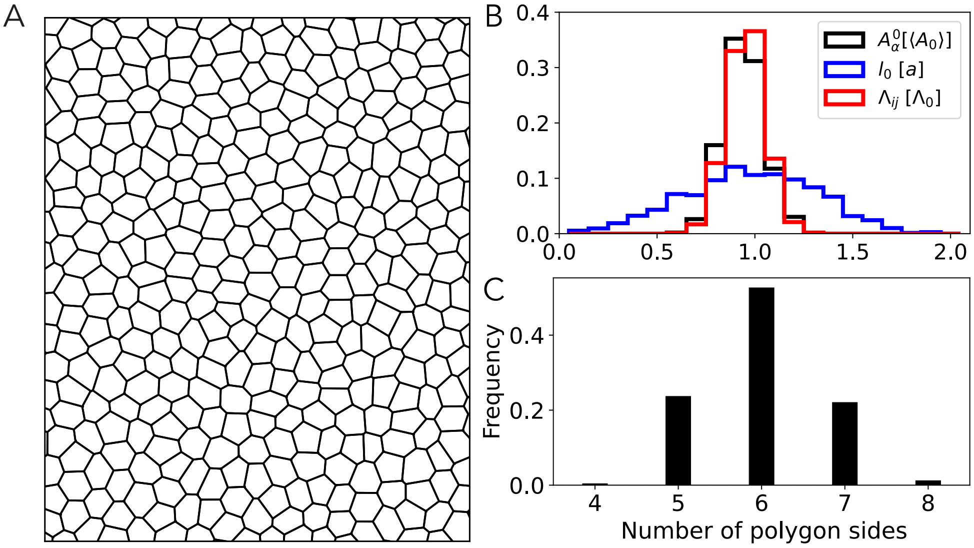

Fig. S1: **Initial configuration of the tissue**. (A) Representative section of the energy relaxed tissue. (B) Histograms of cell target area, junction rest length, and junction tension at the relaxed initial state. Rest length is expressed in units of a, which corresponds to the side of a regular hexagon with area $\langle A_0 \rangle$ $\left(a = 0.62\sqrt{\langle A_0 \rangle}\right)$. (C) Histogram of polygon sidedness in the initial configuration.

1.2 Rules for T1 transitions and higher-order vertex assembly

1. If a junction shared by a n-fold ($n \geq 3$) vertex i and a 3-fold vertex j, with total tension Λ_{ij}, shrinks below a threshold length $l_{ij} < l_{T_1}$, then we remove one of the vertices (j), while transforming the other (i) into a $(n+1)$-fold

vertex. See Fig. S2A-C for examples for $n = \{3,4,5\}$. During this process, each shoulder junction sustaining the $(n+1)$-fold vertex gains $1/(n+1)$ of the tension (Λ_{ij}) in the deleted junction, which remains in the system until the $(n+1)$-fold is resolved. Movie 4 shows a system is which 3-fold, 4-fold and 5-fold vertices are allowed.

Note: In the main text simulations we only show 3-fold and 4-fold vertices. In those simulations, if a junction shared by two vertices i and j shrinks below a threshold $l_{ij} < l_{T_1}$, and at least one of them is a 4-fold vertex, no higher-order vertex is created, and the tension remodeling rate k_C is set to zero until l_{ij} reaches the threshold l_{T_1}.

2. Every τ_{test} timesteps, we attempt to resolve the $(n+1)$-fold vertices present in the tissue. The resolution is tested for each of the $(n+1)$ configurations. During the n-fold vertex resolution timestep, we create a junction of length $1.5 l_{T_1}$ in the direction $(\mathbf{R}_c^\alpha - \mathbf{r}_{n+1}) / \mid \mathbf{R}_c^\alpha - \mathbf{r}_{n+1} \mid$, where $\mathbf{R}_c^\alpha$ is the center of the cell α surrounding the $(n+1)$-fold vertex with position $\mathbf{r}_{n+1}$. See Fig. S2B-D for examples with $n = \{4,5\}$. We assign a tension value Λ_{ij} for the newly created junction drawn from a normal distribution with mean value Λ_0 and standard deviation $0.1\Lambda_0$. Then, the total tension in the new junction is given by $\Lambda_{\text{birth}} = \Lambda_{ij} + 1.5\Gamma_a l_{T_1}$. The rest length l_{ij}^0 of the new junction is drawn from a truncated (only positive values) normal distribution with mean value l_{T_1} and standard deviation equals $0.1 l_{T_1}$. During this process, each shoulder junction sustaining the newly created junction loses $1/(n+1)$ of Λ_{birth}. Finally, we calculate the forces at vertices i and j for each configuration.

   Note: For the 4-fold vertex resolution we combine the four possible configurations into two - the original (reverse T1 transition) and the perpendicular (T1 transition) one. The original one is defined by the direction $\left(\mathbf{R}_c^B - \mathbf{R}_c^D\right) / \mid \mathbf{R}_c^B - \mathbf{R}_c^D \mid$, and the perpendicular by $\left(\mathbf{R}_c^E - \mathbf{R}_c^C\right) / \mid \mathbf{R}_c^E - \mathbf{R}_c^C \mid$, with $\{S,E,B,C\}$ as shown in Fig. S2A.

3. If the effective force between i and j is attractive in all the tested configurations, then the $(n+1)$-fold vertex is considered stable, and we proceed to delete j again. However, if the effective force between i and j is repulsive, at least in one case, then the $(n+1)$-fold vertex is unstable. Therefore, we choose the configuration with the largest repulsive force between the n-fold i and the 3-fold j vertices.

1.3 Choice of model parameters

Our model involves approximately 19 parameters, as listed in Table 1. Many of these parameters are calibrated from prior experimental studies and measurements, several parameters have been varied in our simulations, and the remainder can be eliminated through non-dimensionalization of the equations of motion. Below, we provide a detailed account of our parameter choices.

Within the set of listed parameters, two pertain to defining tissue size (L_x and L_y), one sets the integration time step (Δt), and another establishes the frequency of attempting T1 transitions (τ_{test}). These parameters are essentially model-specific choices, akin to those in any numerical work. Out of the remaining fifteen parameters, three of them (area elastic modulus K, mean preferred area $\langle A_\alpha^0 \rangle$, and friction coefficient μ) have been used to non-dimensionalize the equations of motion. Specifically, we non-dimensionalized force scales by $K(A_\alpha^0)^{3/2}$, length scales by $\sqrt{A_\alpha^0}$, time scales by μ/KA_α^0, setting $K = 1$, $\langle A_\alpha^0 \rangle = 1$, and $\mu = 0.2$ ($\sim 28\,\text{s}$), where $\langle .. \rangle$ represents population average. The positive value for the mean initial tension, $\langle \Lambda_{ij} \rangle = \Lambda_0 = 0.1$, has been chosen to ensure that the initial state of the non-active tissue (further details are available in Section 1.1) resembles a stable, solid-like network [3]. Similar values have been adopted in previous studies that utilized the tension-remodeling model at the junction level to fit experimental data [4, 5, 6].

The tension components arising from contractility (Γ_a) and fluctuations (σ) are assumed to be considerably smaller than the mean initial tension. The relaxation timescale for tension fluctuations is assumed to be twice the friction value, ensuring the persistence of this stochastic term during short time intervals. The longest timescale is attributed to tension relaxation, τ_Λ, which facilitates the dominance of tension remodeling dynamics during the temporal window encompassing 4-fold vertex formation, followed by instantaneous resolution or brief stalling periods.

Out of the remaining seven parameters, four are closely associated with tension remodeling dynamics: strain relaxation rate (k_L), critical strain threshold (ε_c), and two tension remodeling rates (k_E and k_C). For the first two parameters, we have chosen values of similar magnitude as previously benchmarked in references [4, 5], derived from experimental

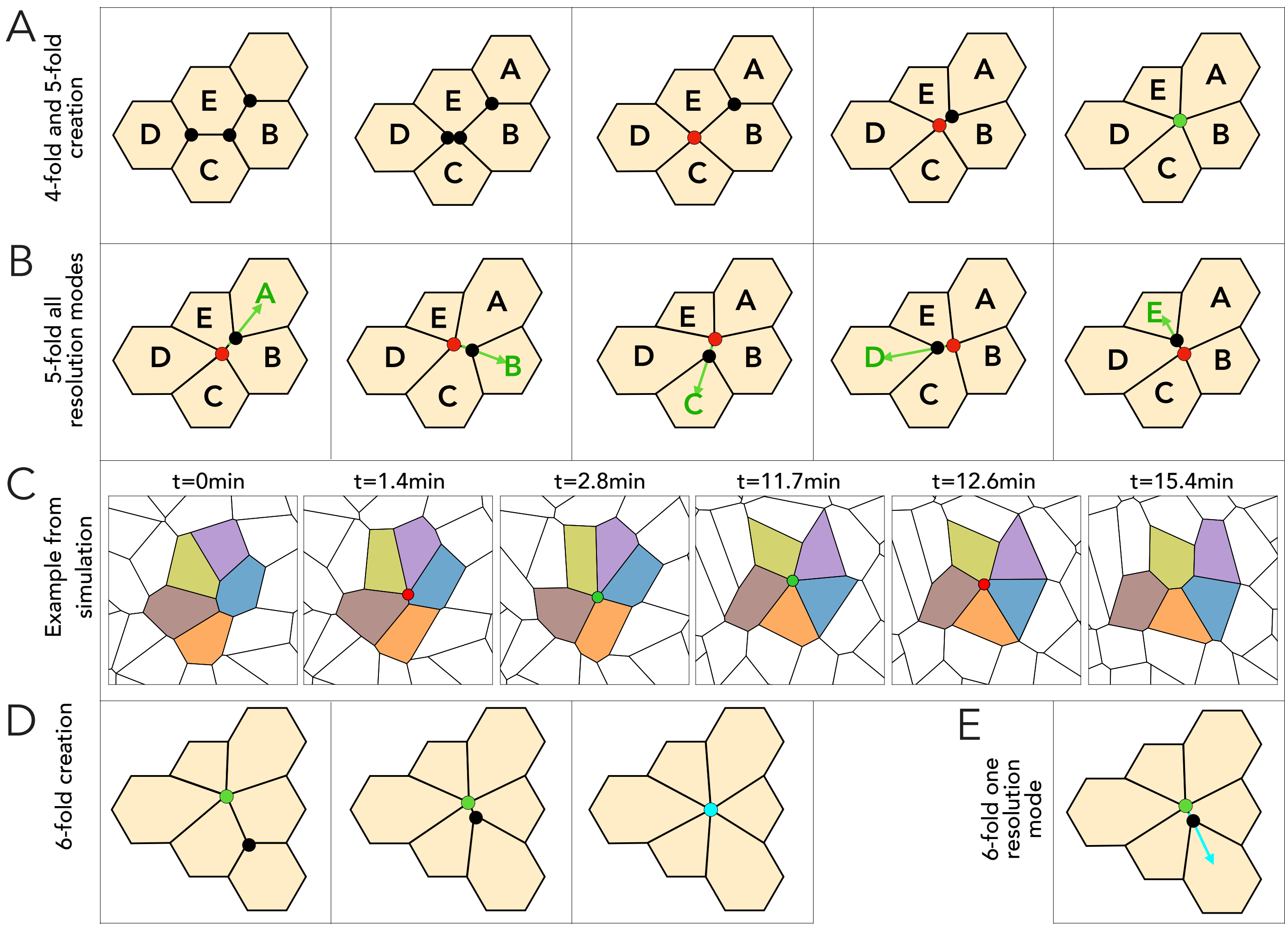

Fig. S2: **Higher-order vertices creation and resolution.** (A) 4-fold and 5-fold creation. (B) Five modes of resolution of a 5-fold vertex. (C) Example of the creation and resolution of a 5-fold vertex (from Movie 4). (D) 6-fold creation from the merging of a 5-fold and a 3-fold vertices. (E) One (out of six) modes of resolution of a 6-fold vertex. Colores circles represent 3-fold (black), 4-fold (red), 5-fold (green), and 6-fold (cyan) vertices.

data. Regarding the tension remodeling rates, we explored values that yield stable systems, as expounded upon in Fig. 1 of the main text.

It is crucial to acknowledge that the values employed in previous studies fall within the range of our examined parameter space. Lastly, we acknowledge that direct experimental data for the tension and length of newly created junctions, as well as the length threshold for 4-fold vertex formation, are exceptionally challenging to obtain due to resolution limitations in current imaging techniques. Nonetheless, we provide informed estimations for these parameters and subsequently vary these three parameters, along with the tension resetting rules during 4-fold creation and resolution, demonstrating that our results remain robust (Figs. S10-S12).

2 Analysis of a positive feedback model between junction tension and strain

In the main text, we studied a negative feedback model between junction tension and strain. Specifically, we used the model that tension increases in junctions under contraction, with a rate proportional to k_C (≥ 0), while it decreases in elongated junctions at a rate proportional to k_E (≥ 0). Here, we analyze the cases where the rates k_C and k_E can take negative values, signifying a positive feedback between tension and strain. We find that transiently stable 4-fold vertices

can arise for $k_C < 0$, if k_E is positive and large enough (Fig. S3). A positive large value for k_E is required to decrease the tension in the extending shoulder junctions compared to the tension in the contracting junction during a T1 event, in order to stabilize a 4-fold vertex. However, with $k_C < 0$, the frequency of T1 events is much lower. For negative values of k_E, all T1 transitions occur instantaneously and 4-fold vertices are unstable.

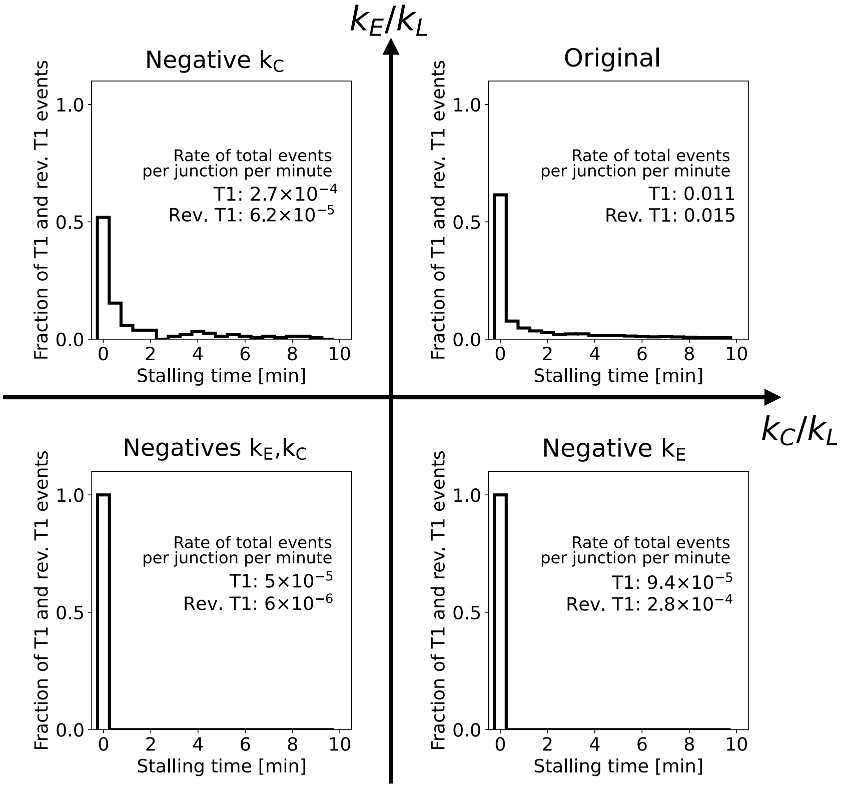

Fig. S3: **Transiently stable 4-fold vertices rely on tension increasing during contraction**. Histograms of the stalling time for T1 and reverse T1 events, for: original simulation ($k_C/k_L = 0.1, k_E/k_L = 0.2$); negative k_C ($k_C/k_L = -0.1, k_E/k_L = 0.2$); negatives k_E, k_C ($k_C/k_L = -0.1, k_E/k_L = -0.2$); and, negative k_E ($k_C/k_L = 0.1, k_E/k_L = -0.2$).

3 Mean-field model

To analytically predict the mechanical stability of cell junctions under contraction, we consider an effective medium theory of the system consisting of two cell junctions in series, connected in parallel to an effective elastic medium of spring constant k (Fig. S4A). Tension in each junction i ($i = 1,2$) is given by Λ_i, with length l_i, rest length l_{0i}, deforming against an overdamped medium with friction coefficient μ. We choose to activate junction 1 with contractility $\Gamma_a > 0$, neglect tension fluctuations, and set the critical strain threshold to 0 for simplicity. Junction tensions and rest lengths evolve following the dynamics defined in main text,

$$\frac{\mathrm{d}\Lambda_1}{\mathrm{d}t} = -k_C(l_1 - l_{01}), \tag{2}$$

$$\frac{\mathrm{d}l_{01}}{\mathrm{d}t} = -k_L(l_{01} - l_1), \tag{3}$$

$$\frac{\mathrm{d}\Lambda_2}{\mathrm{d}t} = -k_E(l_2 - l_{02}), \tag{4}$$

$$\frac{\mathrm{d}l_{02}}{\mathrm{d}t} = -k_L(l_{02} - l_2). \tag{5}$$

Assuming the system conserve its total length, we have the constraint $2L = l_1 + l_2$. Dynamics of junction length then follows from considering the force-balance equation at the vertex between the two junctions,

$$
\begin{aligned}
2\mu \frac{dl_1}{dt} &= k(l_2 - L) + k(L - l_1) + \Lambda_2 - \Lambda_1 - \Gamma_a l_1, \\
&= k(2L - l_1 - L) + k(L - l_1) + \Lambda_2 - \Lambda_1 - \Gamma_a l_1, \\
&= 2k(L - l_1) + \Lambda_2 - \Lambda_1 - \Gamma_a l_1. \qquad (6)
\end{aligned}
$$

3.1 Mechanical stability of cell junctions

The activated junction (junction 1) will collapse to zero length if the system is unstable to contraction. Stability is thus defined by the junction reaching a non-zero length $l_1 > 0$ at steady-state. At steady-state, solution to Eqs. (2)-(6) are given by,

$$0 = 2kL - (2k + \Gamma_a) l_1^{\mathrm{EQ}} + \Lambda_2^{\mathrm{EQ}} - \Lambda_1^{\mathrm{EQ}}, \qquad (7)$$

$$l_{01}^{\mathrm{EQ}} = l_1^{\mathrm{EQ}}, \qquad (8)$$

$$l_{02}^{\mathrm{EQ}} = l_2^{\mathrm{EQ}} = 2L - l_1^{\mathrm{EQ}}. \qquad (9)$$

Since $d\Lambda_1/dt = -(k_C/k_L) dl_{01}/dt$, and $d\Lambda_2/dt = -(k_C/k_L) dl_{02}/dt$, we can integrate these equations from $t = 0$ to the time at which steady-state is reached, to calculate the steady-state tension values Λ_1^{EQ} and Λ_2^{EQ}. Considering that initial lengths and tensions were given by L, and T_0, respectively, we obtain

$$\Lambda_1^{\mathrm{EQ}} = T_0 - \frac{k_C}{k_L}\left(l_{01}^{\mathrm{EQ}} - L\right), \qquad (10)$$

$$\Lambda_2^{\mathrm{EQ}} = T_0 - \frac{k_E}{k_L}\left(l_{02}^{\mathrm{EQ}} - L\right). \qquad (11)$$

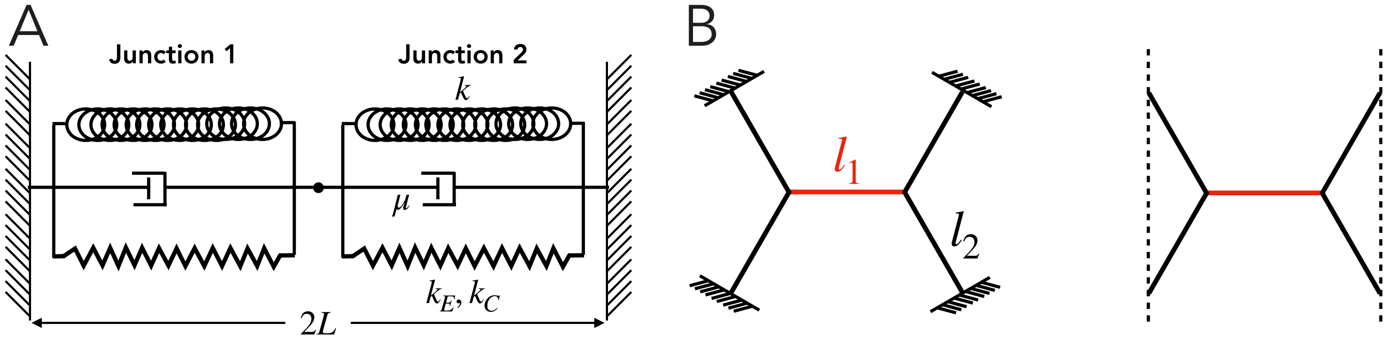

Fig. S4: **Schematic of the mean-field models**. (A) Effective system composed of two junctions of natural length $2L$, under fixed boundary conditions. Each junction is composed of an elastic element with spring constant k, a dashpot with friction coefficient μ, and a tension Λ that remodels at a rate k_C under contraction, and k_E under stretch. Junction rest length remodels at a rate k_L. (B) Schematic of an effective five-junction-system in two dimensions, as part of a hexagonal lattice. The central junction is activated by contraction, sustained by four shoulder junctions. (Left) Shoulder junctions are under a fixed boundary condition. (Right) The four shoulder junctions are free to move vertically.

Using relations in Eqs. (8)-(11) in Eq. (7) we get,

$$
\begin{aligned}
0 &= 2kL-(2k+\Gamma_a)l_1^{\mathrm{EQ}}+T_0-\frac{k_E}{k_L}\left(l_{02}^{\mathrm{EQ}}-L\right)-T_0+\frac{k_C}{k_L}\left(l_{01}^{\mathrm{EQ}}-L\right),\\
0 &= 2kL-(2k+\Gamma_a)l_1^{\mathrm{EQ}}-\frac{k_E}{k_L}\left(2L-l_1^{\mathrm{EQ}}-L\right)+\frac{k_C}{k_L}\left(l_1^{\mathrm{EQ}}-L\right),\\
0 &= 2kL-(2k+\Gamma_a)l_1^{\mathrm{EQ}}-\frac{k_E}{k_L}\left(L-l_1^{\mathrm{EQ}}\right)-\frac{k_C}{k_L}\left(L-l_1^{\mathrm{EQ}}\right),\\
0 &= 2kL-(2k+\Gamma_a)l_1^{\mathrm{EQ}}-\left(\frac{k_E+k_C}{k_L}\right)L+\left(\frac{k_E+k_C}{k_L}\right)l_1^{\mathrm{EQ}},\\
0 &= L\left(2k-\frac{k_E+k_C}{k_L}\right)-\left(2k+\Gamma_a-\frac{k_E+k_C}{k_L}\right)l_1^{\mathrm{EQ}}. \qquad (12)
\end{aligned}
$$

The above equation gives,

$$
l_1^{\mathrm{EQ}} = L\frac{\left(2k-\frac{k_E+k_C}{k_L}\right)}{\left(2k+\Gamma_a-\frac{k_E+k_C}{k_L}\right)}, \qquad (13)
$$

Thus, in order to have $L > l_1^{\mathrm{EQ}} > 0$, $(k_E+k_C)/k_L$ has to be smaller than $2k$.

3.2 Tension change due to remodeling

In the main text, we presented results relating the stability of four-fold vertices to reduction in tension in the tissue. Here we derive the condition for reduction in tension using the effective medium model. We can write Eqs. (10) and (11) in a more general form, with the initial lengths of a junction under contraction (stretch) given by L_C^{ini} (L_E^{ini}),

$$
\Lambda_C^{\mathrm{EQ}} = T_0-\frac{k_C}{k_L}\left(l_{0C}^{\mathrm{EQ}}-L_C^{\mathrm{ini}}\right), \qquad (14)
$$

$$
\Lambda_E^{\mathrm{EQ}} = T_0-\frac{k_E}{k_L}\left(l_{0E}^{\mathrm{EQ}}-L_E^{\mathrm{ini}}\right). \qquad (15)
$$

Assuming that we have A junctions under contraction and B junction under stretch, all of them with initial tension T_0, the global change in tissue tension from an undeformed state is given by

$$
\begin{aligned}
\Delta\Lambda &= \left[AT_0-\frac{k_C}{k_L}\sum_{C=1}^{A}\left(l_{0C}^{\mathrm{EQ}}-L_C^{\mathrm{ini}}\right)+BT_0-\frac{k_E}{k_L}\sum_{E=1}^{B}\left(l_{0E}^{\mathrm{EQ}}-L_E^{\mathrm{ini}}\right)\right]-(A+B)T_0,\\
&= -\frac{k_C}{k_L}\sum_{C=1}^{A}\left(l_{0C}^{\mathrm{EQ}}-L_C^{\mathrm{ini}}\right)-\frac{k_E}{k_L}\sum_{E=1}^{B}\left(l_{0E}^{\mathrm{EQ}}-L_E^{\mathrm{ini}}\right),\\
&= -\left[\frac{k_E}{k_L}\sum_{E=1}^{B}\left(l_{0E}^{\mathrm{EQ}}-L_E^{\mathrm{ini}}\right)-\frac{k_C}{k_L}\sum_{C=1}^{A}\left(L_C^{\mathrm{ini}}-l_{0C}^{\mathrm{EQ}}\right)\right]. \qquad (16)
\end{aligned}
$$

We then define $\delta L^+ = \sum_{E=1}^{B}\left(l_{0E}^{\mathrm{EQ}}-L_E^{\mathrm{ini}}\right)$, and $\delta L^- = \sum_{C=1}^{A}\left(L_C^{\mathrm{ini}}-l_{0C}^{\mathrm{EQ}}\right)$, where δL^+ represents the net elongation of the junctions under stretch, while δL^- represents the net contraction of the junctions under contraction. If $\delta L^+ = \beta\delta L^-$, we then have

$$
\begin{aligned}
\Delta\Lambda &= -\left(\frac{k_E}{k_L}\delta L^+-\frac{k_C}{k_L}\delta L^-\right),\\
&= -\left(\frac{k_E}{k_L}\beta\delta L^--\frac{k_C}{k_L}\delta L^-\right),\\
&= -\frac{\delta L^-}{k_L}\left(\beta k_E-k_C\right), \qquad (17)
\end{aligned}
$$

From the last equation, we can see that the condition for reducing the global tension is $\beta k_E > k_C$, with β depending on the increase or decrease of the total junction length after activation. In a system that conserves its total junction length ($\delta L^+ = \delta L^-$), the condition for reducing the global tension is given by $k_E > k_C$. However, if after the activation, the system increases its total junction length ($\delta L^+ > \delta L^-$), then the condition for reducing tension is given by $\beta k_E > k_C$, with $\beta > 1$. In the context of a confluent tissue simulated with the vertex model, a solid tissue tries to maintain the initial steady-state configuration, and we expect the condition $k_E > k_C$ to hold for global tension reduction. Instead, fluid tissues increase their cell shape index, and then we expect the condition $\beta k_E > k_C$, with $\beta > 1$.

If we now consider a two-dimensional system as in Fig. S4B-(left) and activate the red junction, the system will decrease its total junction length ($\beta < 1$). Instead, if we consider a two-dimensional system as in Fig. S4B-(right), with the condition that outer ends of the shoulder junctions can move vertically while keeping $2l_2 + l_1 = 3L$ fixed, we get

$$\delta L^- = L - l_1, \tag{18}$$

$$\delta L^+ = 4(l_2 - L) = 4(3L/2 - l_1/2 - L) = 2(L - l_1). \tag{19}$$

Thus, $\beta = \delta L^+/\delta L^- = 2$.

4 Additional characterizations of tissue mechanics from the vertex model simulations

Effect of tension remodeling on cellular pressure. Figure S5 shows the distribution of cellular pressure in the tissue, which is given for each cell as $K(A_\alpha - A_\alpha^0)$. The pressure distribution becomes wider and larger in magnitude for higher rates of tension remodeling. This suggests that pressure-like forces play an important role in regulating tissue topology and the stability of four-fold vertices.

Effect of tension remodeling on T1 stalling times. Figure S6 shows the role of tension remodeling on the mean stalling time (in minutes) for T1 transitions. Four-fold vertices are present for longer times for large k_E and small k_C, reaching mean stalling times of 4.5 min and 6 min, for T1 and reversible T1 events, respectively.

Correlation between T1 stalling time and T1 rates. Figure S17 shows the correlation between the rate of T1 transitions per junction and the mean T1 stalling time. The initial increase in the rate of T1 with T1 stalling times indicates that stable four-fold vertices are present in fluid-like tissues with a high rate of T1 events. A negative correlation between the mean stalling time and the rate of T1 events emerges for higher stalling times. The presence of four-fold vertices for longer times implies a decrease in the events of cellular rearrangements.

Role of tension noise amplitude σ. In the main text, all the simulations considered a fixed amplitude of tension fluctuations, $\sigma = 0.02$. Fig. S13A shows how the rate of instantaneous and delayed events change when using different values of the tension noise amplitude σ. We find that tension fluctuations increase the number of T1 events. Additionally, we obtain that T1 stalling time decreases with increasing tension fluctuations (Fig. S13B). Stable four-fold vertices can be present for more than 40 minutes if fluctuations are minimal.

Role of tension relaxation τ_Λ. In the main text, all simulations were run considering a fixed value for the tension relaxation timescale $\tau_\Lambda = 10$. Fig. S14A shows how the rate of instantaneous and delayed events change when using different values of τ_Λ. Larger tension relaxation timescales decrease the number of T1 events, while increasing T1 stalling times (Fig. S14B).

Finite shear simulations over solid and fluid tissues. We perform external finite shear over tissues defined as solid and fluid by cell's center diffusion, with (Fluid II) and without (Fluid I) transiently stable 4-fold vertices, Fig. S16. The relaxation scale for the solid tissue is of hours, while for the fluid tissues is of minutes. Tissue Fluid-II releases energy and reduces stress faster than Tissue Fluid-I, even though it has fewer instantaneous T1 events, or instantaneous cell neighbor exchanges. We find that active tension remodeling in addition to mechanical memory loss mechanisms develop transiently stable rosettes in Fluid-II, generating a mechanical material that responds more liquid-like than Fluid-I.

5 Analysis of different rules during the creation and resolution of 4-fold vertices

Effect of varying the critical strain parameter ε_c. The threshold strain for tension remodeling is motivated by experimental data on single junction activation. However, we can remove this parameter from our model Fig. S7 by setting $\varepsilon_c = 0$. We find that the absence of threshold strain does not affect the role of junction tension remodeling in controlling the rates of T1 transitions. Additionally, we find that varying the value of ε_c does not induce major changes in the probabilities of delayed events nor the distribution of T1 stalling times, Fig. S12.

No change in shoulders tension and uniform Λ_{birth}. In the main text, all simulations were run considering a gain and loss in the shoulders tension during the creations and resolution of 4-fold vertices, respectively, motivated by experimental observations of Myosin-II accumulation around junctions proximal to 4-fold vertices. Here we present simulations results assuming no change in the shoulder tension, and $l^0_{\text{birth}} = l_{\text{birth}} = 1.5 l_{T1}$ (simpler rules). First we use $\Lambda_{\text{birth}} = \Lambda_0$ (mean initial tension), Fig. S8. We obtain the same qualitative results (three regions: quiescent, instantaneous events, delayed events), with larger probabilities of transiently stable 4-fold vertices. Then, considering a small new tension $\Lambda_{\text{birth}} = 0.1\Lambda_0$, Fig. S9 we obtained the same previous results. However, differently from the original simulations, under these rules the probability of stable 4-fold vertices increases with both k_C/k_L and k_E/k_L. Particularly for $k_C/k_L = 0.17, k_E/k_L = 0.20$, we quantified the stalling times associated to delayed T1 and reverse T1 events for the different models, including the case of $\Lambda_{\text{birth}} = 0$, Fig. S10. Interestingly, even for $\Lambda_{\text{birth}} = 0$ we obtain delayed events. However, we find that the probability and stalling time decrease with smaller Λ_{birth}.

Effect of different l_{birth} on T1 and reverse T1 stalling times. In the main text, all simulations were run considering that right after resolution a new junction has a length $l_{\text{birth}} = 1.5 l_{T_1}$. We run simulations for $k_C/k_L = 0.17, k_E/k_L = 0.20$, considering $l_{\text{birth}}/l_{T_1} = \{1.1, 1.3, 1.5, 1.7\}$. We do not observe any major changes on the probabilities of delayed events nor on the stalling times, Fig. S11.

Effect of persistent-tension rule. We ran a simulation ($k_C/k_L = 0.1, k_E/k_L = 0.2$) considering a persistent-tension rule, i.e., a newly birth junction recovers the tension it used to have before forming the 4-fold vertex. We find the emergence of permanently stable 4-fold vertices in this case, as shown in Fig. S15.

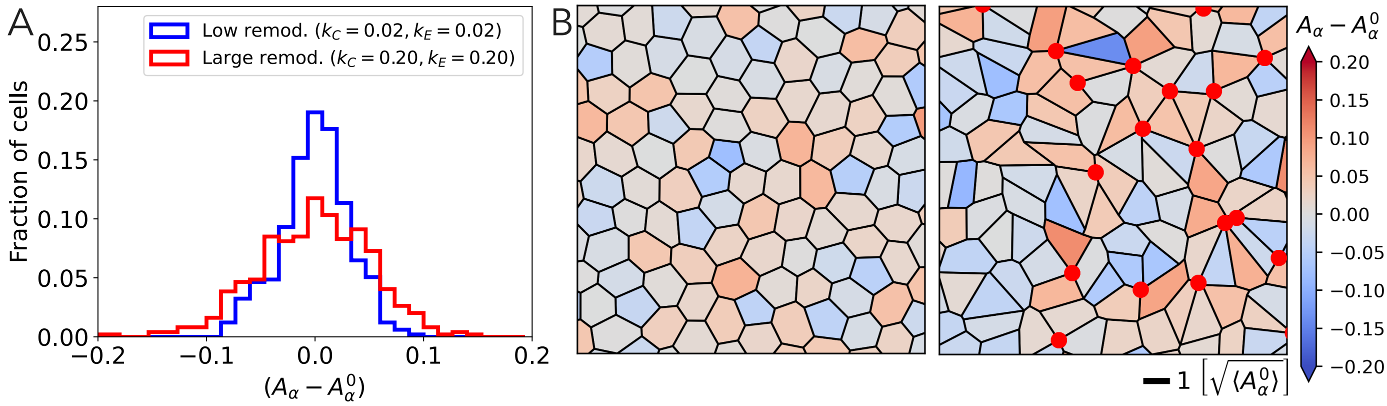

Fig. S5: **Role of tension remodeling on the distribution of cellular pressure**. (A) Histogram of cellular pressure for low ($k_C/k_L = k_E/k_L = 0.02$) and higher ($k_C/k_L = k_E/k_L = 0.20$) rates of tension remodeling. (B) Snapshots of the tissues considered in (A) show the spatial distribution of cellular pressure, for low (left) and high (right) rates of tension remodeling. Red circles represent four-fold vertices.

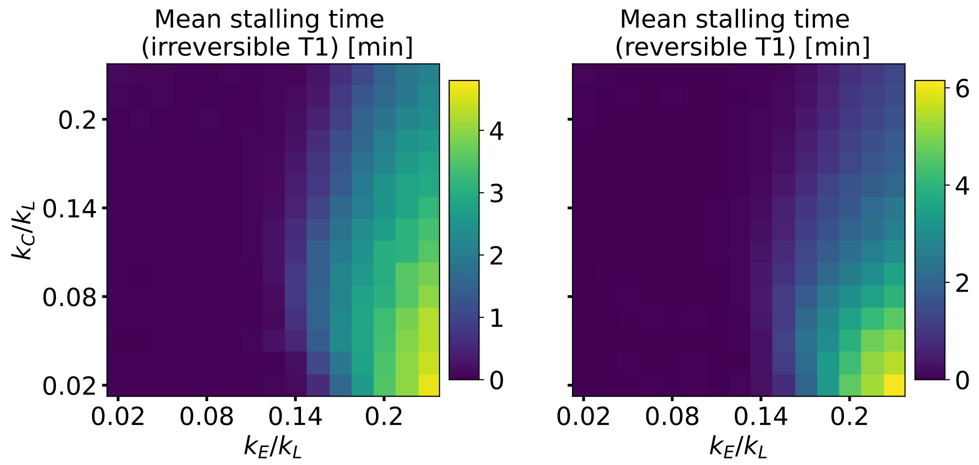

Fig. S6: Mean T1 stalling time (colorscale) as functions of normalized tension remodeling rates, k_E/k_L (x-axes) and k_C/k_L (y-axes). Left: Mean stalling time for irreversible T1 transitions. Right: Mean stalling time for reversible T1 transitions.

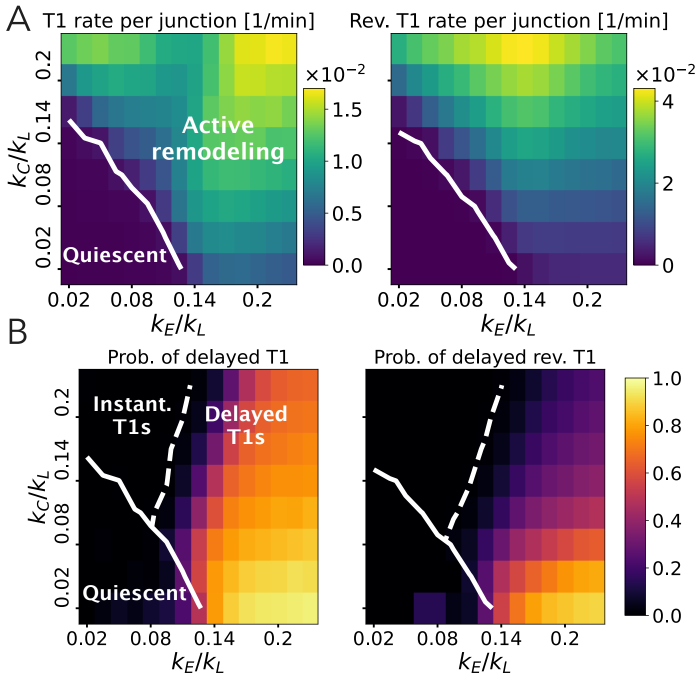

Fig. S7: **Phase diagrams with no critical strain ($\varepsilon_c = 0$) for tension remodeling**. (A) Rates of T1 (left) and reversible (right) transitions for different values of k_E/k_L and k_C/k_L. Solid lines represent 10^{-3} T1 events per junction per minute. (B) Probability of stalled/delayed irreversible T1 transitions (left) and reversible T1 events (right), for different values of k_E/k_L and k_C/k_L. Dashed lines represent 1% probability.

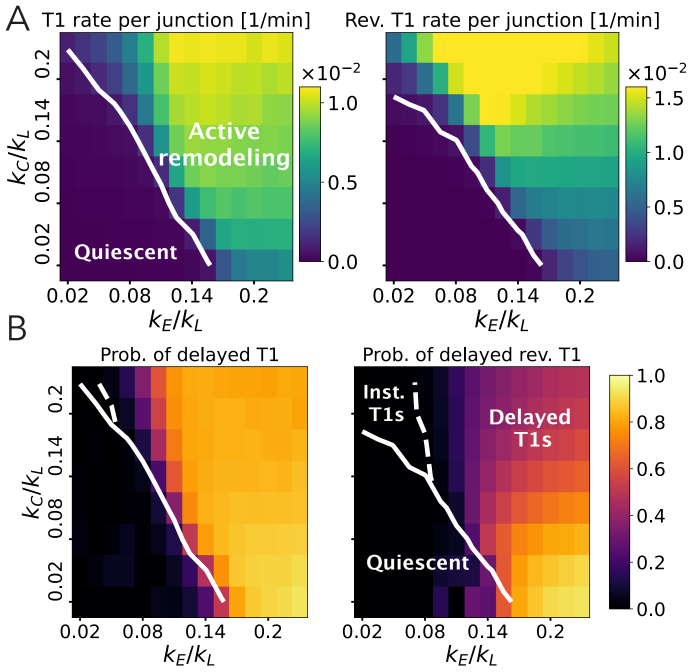

Fig. S8: **Phase diagrams with a constant tension after T1 transition, $\Lambda_{\mathbf{birth}} = \Lambda_0$.** (A) Rates of T1 (left) and reversible (right) transitions for different values of k_E/k_L and k_C/k_L. Solid lines represent 10^{-3} T1 events per junction per minute. (B) Probability of stalled/delayed irreversible T1 transitions (left) and reversible T1 events (right), for different values of k_E/k_L and k_C/k_L. Dashed lines represent 1% probability. For these simulations we consider $\Lambda_{\text{birth}} = \Lambda_0$ (no noise on the tension of the newly born junction).

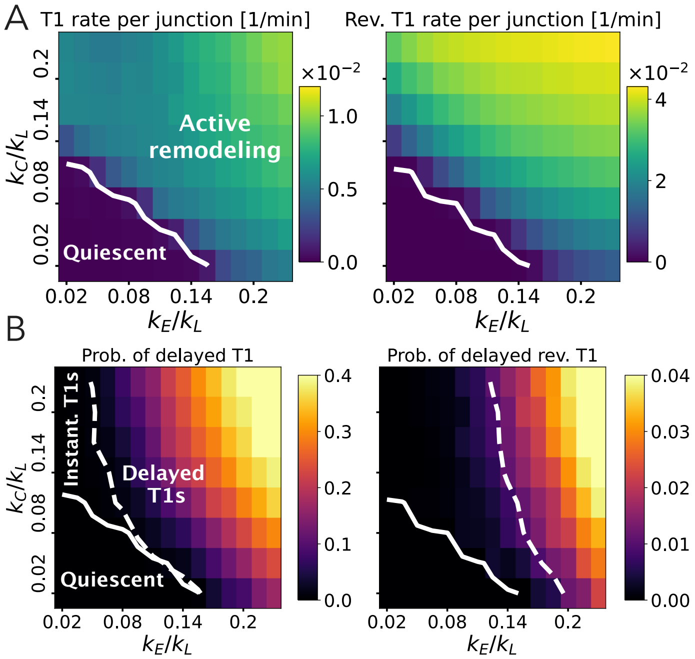

Fig. S9: **Phase diagrams with a reduced tension after T1 transition, $\Lambda_{\mathbf{birth}} = 0.1\Lambda_0$.** (A) Rates of T1 (left) and reversible (right) transitions for different values of k_E/k_L and k_C/k_L. Solid lines represent 10^{-3} T1 events per junction per minute. (B) Probability of stalled/delayed irreversible T1 transitions (left) and reversible T1 events (right), for different values of k_E/k_L and k_C/k_L. Dashed lines represent 1% probability. For these simulations we consider no change in shoulder junctions tension during the creation and resolution of 4-fold vertices, and $\Lambda_{\text{birth}} = 0.1\Lambda_0$ (no noise on the small tension of the newly born junction).

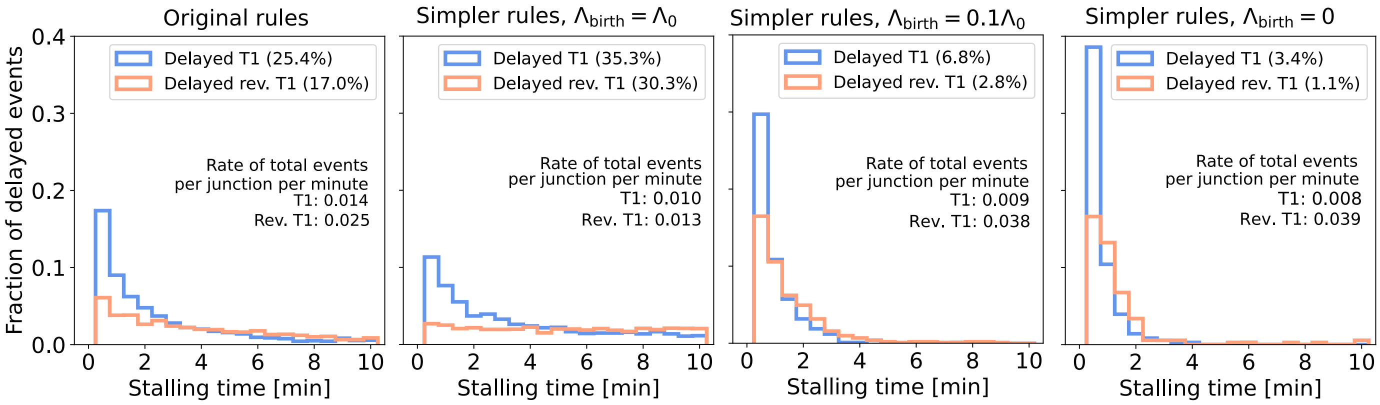

Fig. S10: Comparison of histograms of the stalling time for delayed irreversible T1 (blue) and delayed reversible T1 (red) events, for $k_C/k_L = 0.17, k_E/k_L = 0.20$, when considering different tension resetting rules after a T1 transition. Simpler rules: no change in shoulder junctions tension during the creation and resolution of 4-fold vertices, and zero birth junction strain ($l^0_{\text{birth}} = l_{\text{birth}} = 1.5 l_{T1}$).

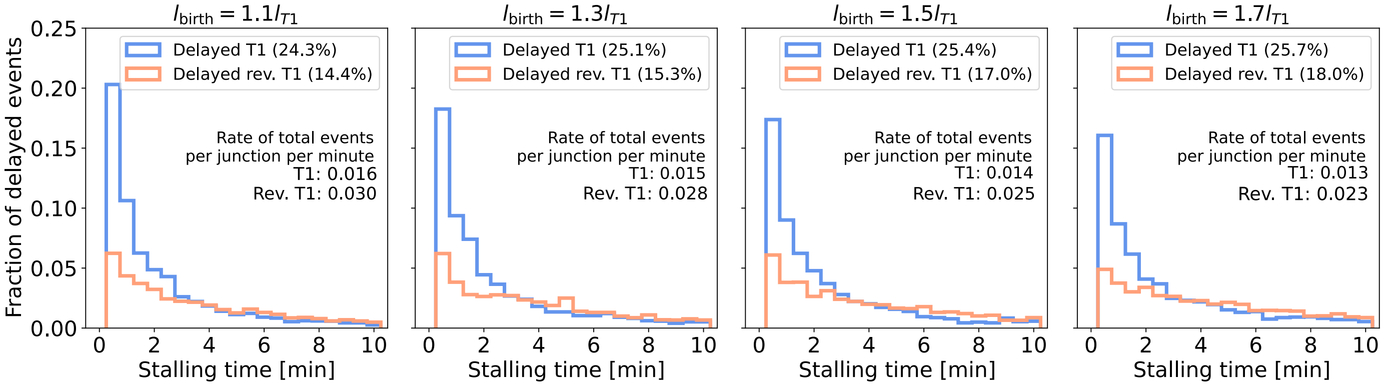

Fig. S11: Comparison of histograms of the stalling time for delayed irreversible T1 (blue) and delayed reversible T1 (red) events, for $k_C/k_L = 0.17, k_E/k_L = 0.20$, when considering different values l_{birth} (junction length after a T1 transition).

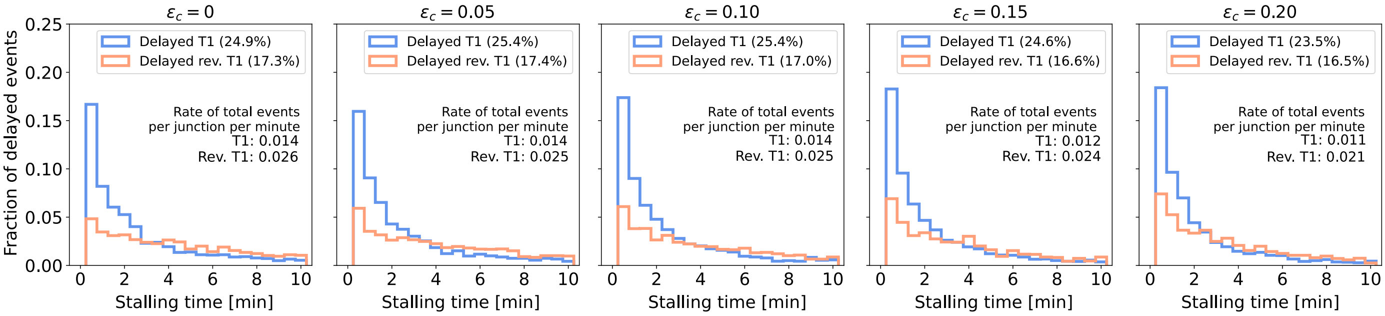

Fig. S12: Comparison of histograms of the stalling time for delayed irreversible T1 (blue) and delayed reversible T1 (red) events, for $k_C/k_L = 0.17, k_E/k_L = 0.20$, when considering different values of the critical strain threshold, ε_c.

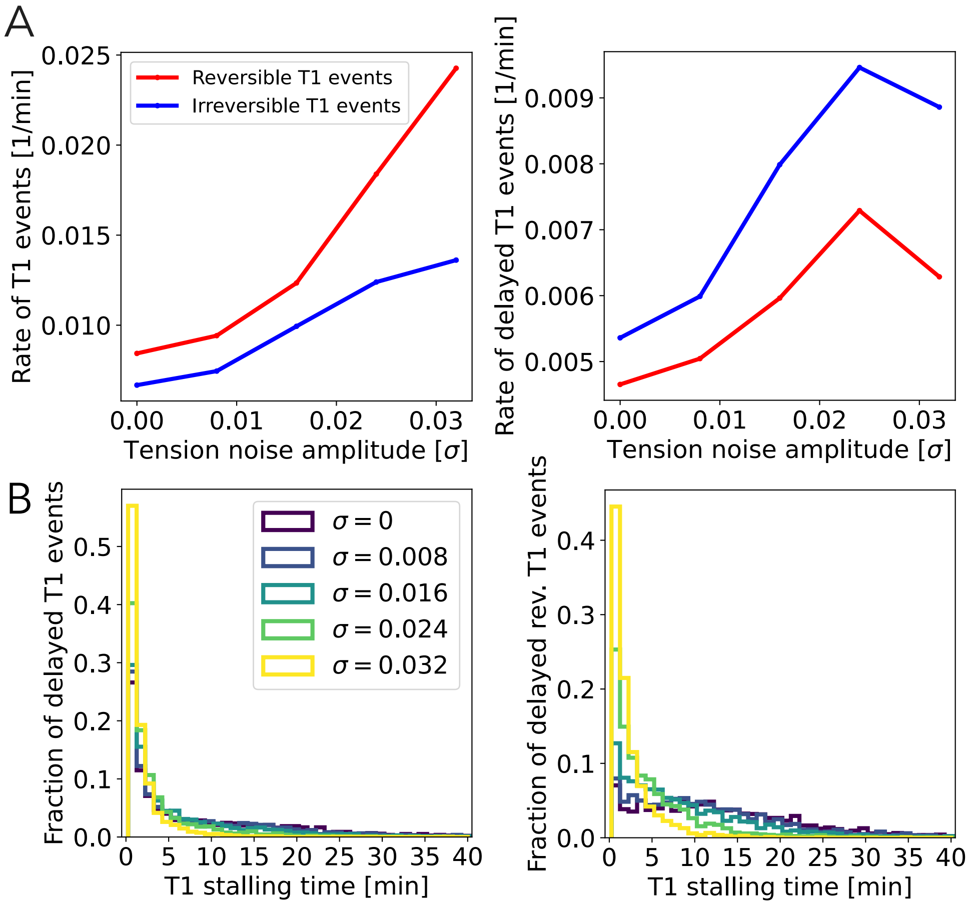

Fig. S13: **Role of tension fluctuations on the rate of T1 events and T1 stalling times**. (A) Rate of irreversible and reversible T1 events (left: all T1 events, right: only delayed T1 events), for $(k_C/k_L = 0.1, k_E/k_L = 0.2)$ (fluid tissue) and different values of the tension noise amplitude σ. (B) Histogram of T1 stalling times for delayed irreversible T1 events (left) and delayed reversible T1 events (right), for different values of the tension noise amplitude σ.

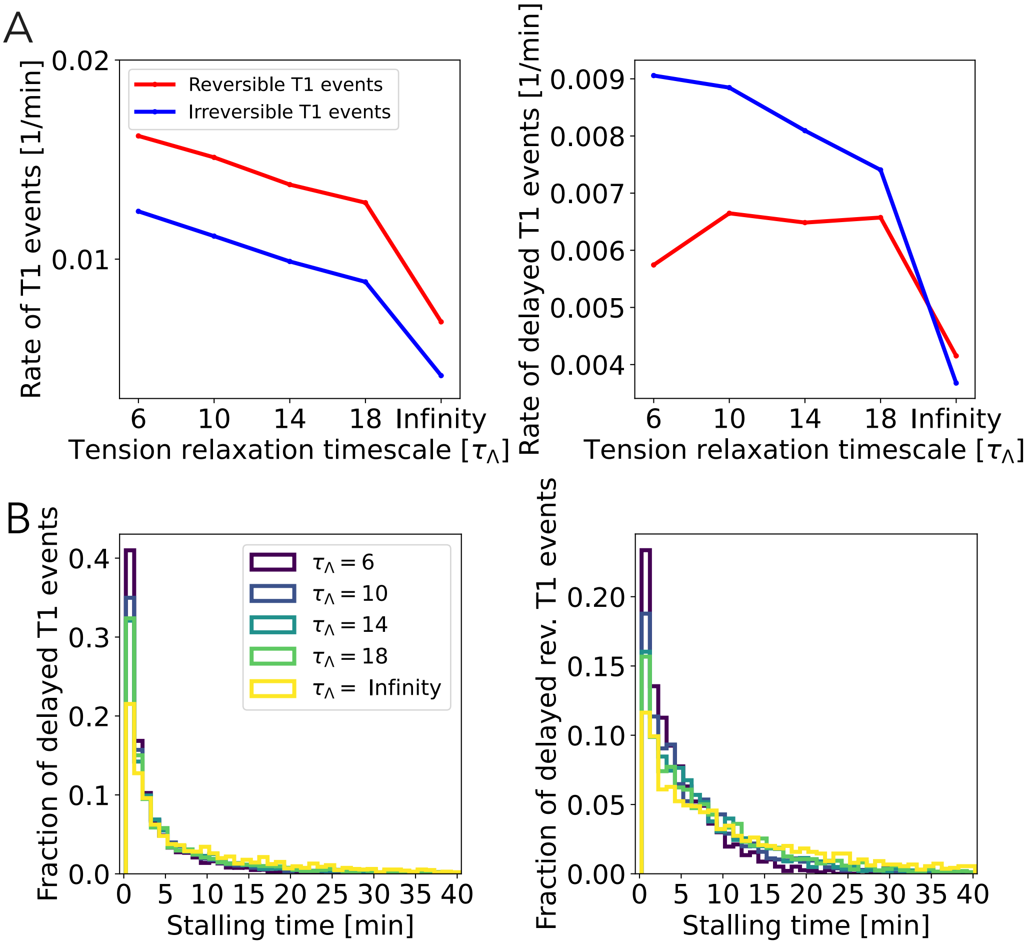

Fig. S14: **Role of the tension relaxation timescale on the rate of T1 events and T1 stalling times**. (A) Rate of irreversible and reversible T1 events (left: all T1 events, right: only delayed T1 events), for $(k_C/k_L = 0.1, k_E/k_L = 0.2)$ (fluid tissue) and different values of the tension relaxation timescale τ_Λ. (B) Histogram of T1 stalling times for delayed irreversible T1 events (left) and delayed reversible T1 events (right), for different values of τ_Λ.

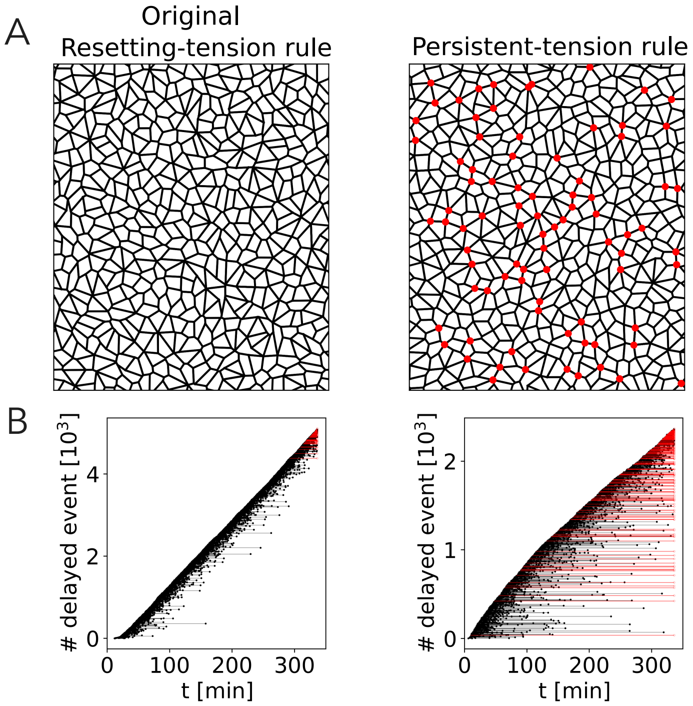

Fig. S15: Comparison of T1 resolution times for delayed T1 events, for an active tissue with $k_C/k_L = 0.1$, $k_E/k_L = 0.20$, with (left column) tension-resetting model and (right column) a persistent tension model during 4-fold vertex resolution. (A) Tissue configurations showing the steady-state morphology (at $\sim 350\,\text{min}$), where red solid circles represent 4-fold vertices that have been stable for more than $100\,\text{min}$ by the end of each simulation. (B) Each line represents the creation of a 4-fold vertex. Black lines represent 4-fold vertices that are resolved through the simulation (at the time highlighted by a black dot), in a time larger than 6 seconds. Red lines, that finish in an empty red circle, represent 4-fold vertices that are not resolved during the simulation.

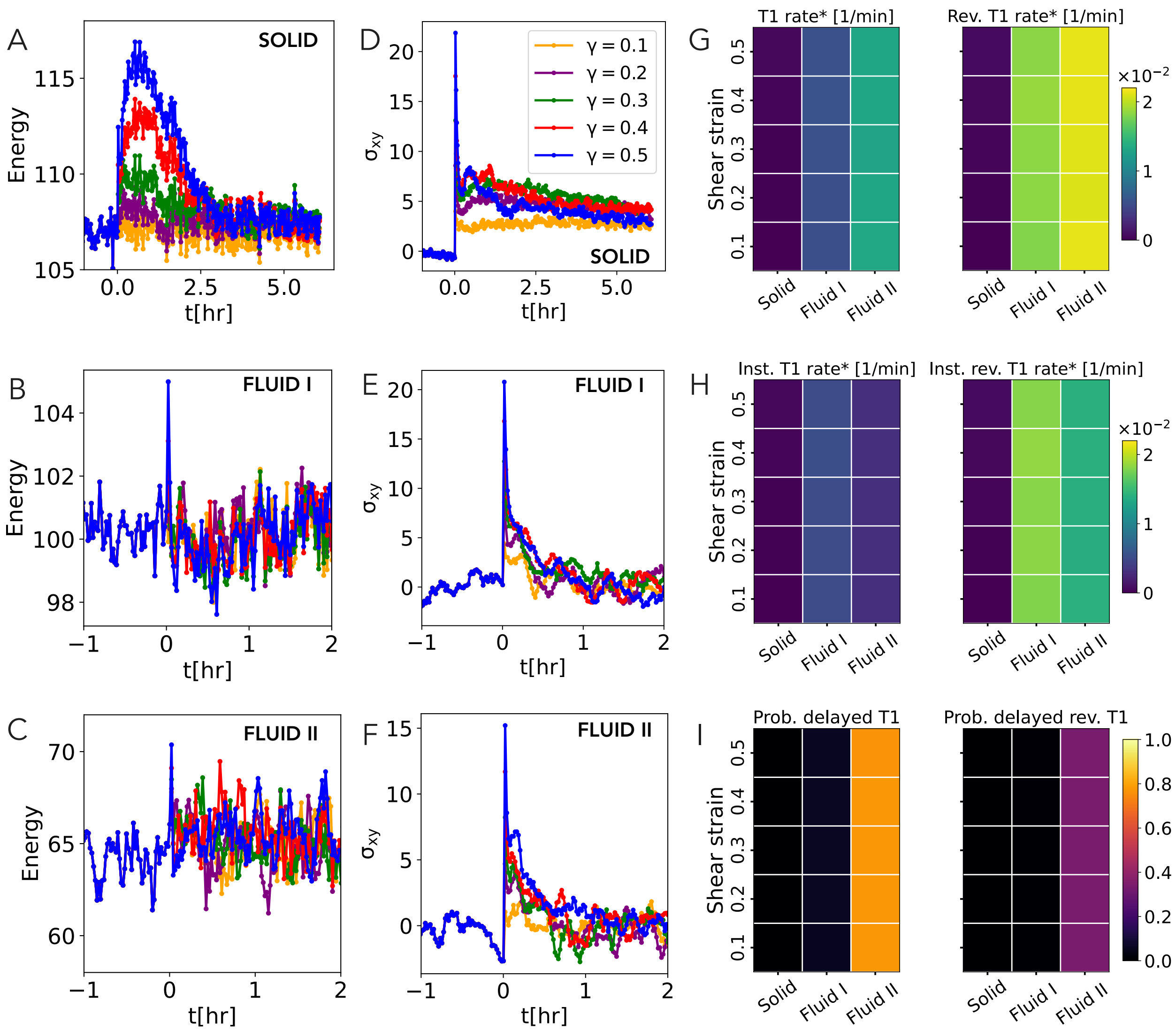

Fig. S16: **Tissue response upon finite shear simulations**. Mechanical response (A-C: energy and D-F: stress release) under finite shear $\gamma = \{0.1, 0.2, 0.3, 0.4, 0.5\}$, applied at $t = 0$, considering Edwards periodic boundary conditions, on three tissues with $k_C/k_L = 0.14$: Solid ($k_E/k_L = 0.05$), Fluid I ($k_E/k_L = 0.11$, without transiently stable 4-fold vertices), and Fluid II ($k_E/k_L = 0.20$, with transiently stable 4-fold vertices). Here, energy is defined as $E_{\mathrm{el}} + (T_{ij} + \Gamma_a l_{ij}/2)\, l_{ij}$, and stress as $\sigma_{xy} = \sum_{i,j} l_{ij}^x l_{ij}^y \,(T_{ij} + \Gamma_a l_{ij})\,/l_{ij}$. G: rate of T1 and reverse T1 events (* means normalized by 1482 junctions). H: rate of instantaneous events (* means normalized by 1482 junctions). I: Probability of delayed events.

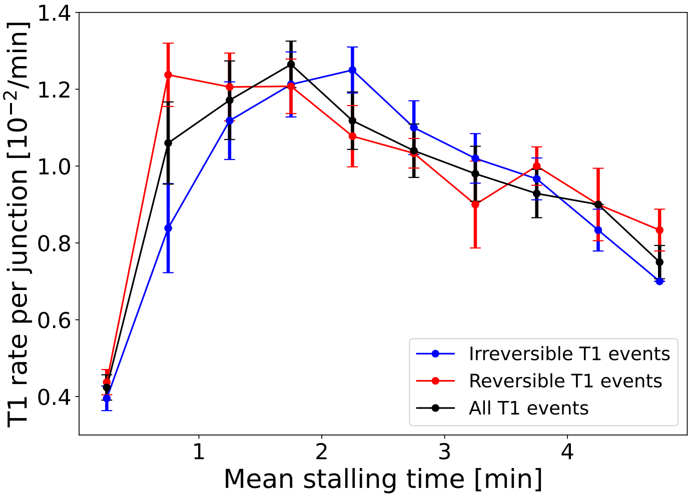

Fig. S17: Correlation between the rate of T1 transitions and the mean T1 stalling times. A negative correlation emerges at larger stalling times. Error bars represent ±1 standard error of mean.

References

[1] F. Pérez-Verdugo, J.-F. Joanny, and R. Soto, "Vertex model instabilities for tissues subject to cellular activity or applied stresses," *Physical Review E*, vol. 102, no. 5, p. 052604, 2020.

[2] D. Bi, J. Lopez, J. M. Schwarz, and M. L. Manning, "A density-independent rigidity transition in biological tissues," *Nature Physics*, vol. 11, no. 12, pp. 1074–1079, 2015.

[3] R. Farhadifar, J.-C. Röper, B. Aigouy, S. Eaton, and F. Jülicher, "The influence of cell mechanics, cell-cell interactions, and proliferation on epithelial packing," *Current Biology*, vol. 17, no. 24, pp. 2095–2104, 2007.

[4] M. F. Staddon, K. E. Cavanaugh, E. M. Munro, M. L. Gardel, and S. Banerjee, "Mechanosensitive junction remodeling promotes robust epithelial morphogenesis," *Biophysical Journal*, vol. 117, no. 9, pp. 1739–1750, 2019.

[5] K. E. Cavanaugh, M. F. Staddon, E. Munro, S. Banerjee, and M. L. Gardel, "Rhoa mediates epithelial cell shape changes via mechanosensitive endocytosis," *Developmental Cell*, vol. 52, no. 2, pp. 152–166, 2020.

[6] K. Nishizawa, S.-Z. Lin, C. Chardès, J.-F. Rupprecht, and P.-F. Lenne, "Two-point optical manipulation reveals mechanosensitive remodeling of cell–cell contacts in vivo," *Proceedings of the National Academy of Sciences*, vol. 120, no. 13, p. e2212389120, 2023.
